# SQuIRE reveals locus-specific regulation of interspersed repeat expression

**DOI:** 10.1093/nar/gky1301

**Published:** 2019-01-09

**Authors:** Wan R Yang, Daniel Ardeljan, Clarissa N Pacyna, Lindsay M Payer, Kathleen H Burns

**Affiliations:** 1 Department of Pathology, Johns Hopkins University School of Medicine, Baltimore, MD 21205, USA; 2 McKusick-Nathans Institute of Genetics, Johns Hopkins University School of Medicine, Baltimore, MD 21205, USA; 3 Thomas C. Jenkins Department of Biophysics, Johns Hopkins University, Baltimore, MD, USA; 4 Sidney Kimmel Comprehensive Cancer Center, Johns Hopkins University School of Medicine, Baltimore, MD, USA

## Abstract

Transposable elements (TEs) are interspersed repeat sequences that make up much of the human genome. Their expression has been implicated in development and disease. However, TE-derived RNA-seq reads are difficult to quantify. Past approaches have excluded these reads or aggregated RNA expression to subfamilies shared by similar TE copies, sacrificing quantitative accuracy or the genomic context necessary to understand the basis of TE transcription. As a result, the effects of TEs on gene expression and associated phenotypes are not well understood. Here, we present Software for Quantifying Interspersed Repeat Expression (SQuIRE), the first RNA-seq analysis pipeline that provides a quantitative and locus-specific picture of TE expression (https://github.com/wyang17/SQuIRE). SQuIRE is an accurate and user-friendly tool that can be used for a variety of species. We applied SQuIRE to RNA-seq from normal mouse tissues and a *Drosophila* model of amyotrophic lateral sclerosis. In both model organisms, we recapitulated previously reported TE subfamily expression levels and revealed locus-specific TE expression. We also identified differences in TE transcription patterns relating to transcript type, gene expression and RNA splicing that would be lost with other approaches using subfamily-level analyses. Altogether, our findings illustrate the importance of studying TE transcription with locus-level resolution.

## INTRODUCTION

Transposable elements (TEs) are self-propagating mobile genetic elements. Their insertions have resulted in a complex distribution of interspersed repeats comprising almost half of the human genome (1,2). However, most TEs have lost the capacity for generating new insertions over their evolutionary history and are now fixed in the human population. Nevertheless, even elements that have lost the potential to retrotranspose can still be transcribed from their locations in the genome. TEs are significant contributors of promoters (3–5) and *cis*-regulatory elements (6–14). Transcription of TEs has been implicated in physiological processes in development and early embryonic pluripotency (15,16). Conversely, TE expression can also be subject to transcriptional silencing (17–21). Loss of these regulatory mechanisms resulting in aberrant TE expression has been associated with cancer (22–24), neurodegenerative diseases (25–29), and infertility (30–33). However, a deeper understanding of how TE transcription impacts these biological processes has been limited by difficulties analyzing TE transcription in RNA sequencing (RNA-seq) data.

TEs propagate using either DNA (‘transposons’) or RNA intermediates (‘retrotransposons’) (34,35). Retrotransposons are further classified into Orders, namely long terminal repeats (LTR), long interspersed elements (LINEs), and short interspersed elements (SINEs) (36). Most elements in animal genomes have accumulated nucleotide substitutions over millions of years. However, a subset remain retrotranspositionally active and generate new polymorphic insertions (37,38). The lack of unique sequence, particularly in newer TE insertions, has presented a problem for short-read RNA sequencing (39). Due to the repetitive nature of TEs, RNA-seq reads that originate from one locus can ambiguously align to many TEs sharing similar sequence dispersed throughout the genome. Because of these barriers, conventional RNA-seq analyses of TEs have either discarded multi-mapping alignments (10) or combined TE expression to the subfamily level (40–42). Other groups have studied active LINE-1s using tailored pipelines, leveraging internal sequence variation and 3′ transcription extensions into unique sequence (43–45). However, these targeted approaches do not provide a global picture of expression from all classes of TEs. TE studies done at the subfamily level are unable to distinguish *TE-intrinsic* expression via TE-derived regulatory sequences from *TE-extrinsic* expression due to TE inclusion in a longer transcript. Conversely, RNA-seq pipelines that rely solely on uniquely aligning reads can miss differential expression of highly repetitive TEs. There is thus a need for locus-specific analyses of TE expression to understand their regulation in normal and disease states.

To analyze global TE expression in conventional RNA-seq experiments, we have developed the Software for Quantifying Interspersed Repeat Expression (SQuIRE). SQuIRE provides a suite of tools to ensure the pipeline is user-friendly, reproducible, and broadly applicable. Like previous TE expression software, SQuIRE quantifies expression at the subfamily level and performs differential expression analyses on TEs and genes. Unlike past approaches however, SQuIRE quantifies TE expression at the locus level. We benchmarked this pipeline using both simulated and experimental datasets and compared its performance against other software to quantify TE expression (40–42). We applied SQuIRE to mouse tissue RNA-seq data and identified examples of locus-specific differential expression in testis compared to somatic tissues. SQuIRE enabled us to assess the physical context of expressed TEs (i.e. the location of these TEs in the genome and within potentially larger RNA transcripts). This revealed many examples of extrinsic regulation of TE expression as part of long transcripts, which subfamily-level analyses would otherwise miss. We also identify specific differentially expressed endogenous retroviral *Gypsy* loci in a *Drosophila* model of amyotrophic lateral sclerosis (ALS) (46). Our findings confirm that locus-specific analysis, attainable with SQuIRE, is essential to get a true picture of the TE transcriptome.

## MATERIALS AND METHODS

### Software and implementation

SQuIRE was written in Python 2 and tested with the following specific versions of software: STAR 2.5.3a (47), BEDtools 2.25.0 (48), SAMtools 1.8 (49), StringTie 1.3.3b (50), DESeq2 1.16.1 (51), R 3.4.1 (52) and Python 2.7.9. SQuIRE was developed for UNIX environments. Briefly, the SQuIRE pipeline includes **Fetch** to obtain reference annotation files, **Map** to align RNA-seq data, **Count** to quantify gene and TE expression, and **Call** to perform differential analysis. The algorithm for quantifying TE expression is exclusive to SQuIRE and described below. Details of the software parameters implemented in the SQuIRE pipeline are described in Supplementary Methods. We provide step-by-step instructions on our README to use the package manager Conda (conda.io) to download the correct versions of prerequisite software for SQuIRE (e.g. Python, R (52), STAR, BEDTools, StringTie, SAMtools, DESeq2). The README also instructs users how to create a non-reference table with the exogenous or polymorphic TE sequences and coordinates that they would like to add to the reference genome. Bash scripts to run each tool in the SQuIRE pipeline are also available on the website. Users can fill in crucial experiment information (raw data, read length, paired, strandedness, genome build, sample name and experimental design) into the ‘arguments.sh’ file, which the other scripts reference to run each step with the correct parameters.

### Quantification algorithm

To quantify TE expression, **Count** first identifies reads that map to TEs. If a TE-mapping read aligns to a single locus after a genome-wide scan, it is labeled as a ‘unique read’; if the read maps to multiple locations, it is labeled as a ‘multi-mapped read’. Count allows for 50% of the read to map to flanking sequence to increase the detection of uniquely aligning reads. For paired-end reads, each individual end is first assessed for unique alignment before identifying their mates. If one multi-mapping end is paired with a uniquely aligning mate, the pair is considered ‘unique’ and other alignments of the multi-mapping mate are discarded. If the RNA-seq data is stranded, the sense and anti-sense direction of a TE are treated as separate transcripts to which a read can align. Second, **Count** assigns fractions of a read to each TE as a function of the probability that the TE gave rise to that read. Uniquely aligning reads are considered certain (i.e. probability = 100%, count = 1). **Count** initially assigns fractions of multi-mapping reads to TEs in proportion to their relative expression as indicated by unique read alignments. In doing so, **Count** also considers that TEs have varying uniquely alignable sequence lengths. To mitigate bias against the *n* number of TEs without uniquely aligning reads, these TEs receive fractions inversely proportional to the number of loci (*N*) to which each read aligned. Then **Count** assigns the remainder $( {1 - \frac{n}{N}} )$ to the TEs with unique reads. If both mates of a read pair are multi-mapping, but only map concordantly to a single TE location, the discordant alignments are discarded. The read pair contributes a full read count to the TE, but they are not considered ‘unique’ and their positions do not contribute to the TE’s uniquely alignable length. To account for TEs that have fewer unique counts due to having less unique sequence, **Count** normalizes each unique count (${C_U}$) to the number of individual unique read start positions, or each TE’s uniquely alignable length (${L_U}$). Among all TEs to which a multi-mapping read aligned, the TEs with unique reads ($s \in T)$ are compared with each other. A fraction of a read is assigned to each TE in proportion to the contribution of the normalized unique count ($\frac{{{C_U}}}{{{L_U}}})$ to the combined normalized unique count of all of the TEs being compared ($\mathop \sum \limits_{s \in T} \frac{{Cs}}{{{L_s}}})$ (Equation 1). Thus, the sum of unique counts and multi-mapped read fractions for each TE provides an initial estimate of TE read abundance based on empirically obtained unique read counts and uniquely alignable sequence.

(1) \begin{equation*}f_{TE}^r = \frac{{\frac{{{C_U}}}{{{L_U}}}}}{{\mathop \sum \nolimits_{s \in T} \frac{{Cs}}{{{L_s}}}}}\ \ \times \ \left( {1 - \frac{n}{N}} \right)\end{equation*}

At this point, multi-mapping reads are assigned to TEs with no unique reads based only on the numbers of valid alignments for each read. This can result in over- or under-estimations of TE expression. To combat this issue, **Count** next refines this initial assignment by redistributing multi-mapping read fractions in proportion to estimated TE expression with an expectation-maximization algorithm. To estimate expression, **Count** uses the a TE’s total read count (${C_{TE}}$ = unique read counts + multi-mapped fractions from the previous step) normalized by the effective transcript length (${l_{TE}}$): $\frac{{{C_{TE}}}}{{{l_{TE}}}}.$ The effective transcript length ${l_{TE}}$ is calculated as the estimated transcript length ${L_{TE}}$ subtracted by the average fragment length aligned to that TE + 1, (${l_{TE}} = {L_{TE}}\ - {l_{avg}} + 1$), as described previously (53). All of the TEs to which a multi-mapping read aligned ($s \in T)$ are compared with each other. A fraction of a read is assigned to each TE in proportion to the relative normalized total count ($\frac{{{C_{TE}}}}{{{l_{TE}}}}$) compared to the combined normalized total count of all of the TEs being compared ($\mathop \sum \limits_{s \in T} \frac{{{T_s}}}{{{l_s}}})$, as shown in Equation (2). **Count** assumes this value is proportional to the probability that the TE gave rise to the multi-mapping read, and assigns that fraction of a read count to the TE. Because TEs with a count fraction of less than 1 have a low probability of giving rise to any read, those TEs are assigned a count fraction of 0.

(2) \begin{equation*}f_{TE}^r = \frac{{\frac{{{C_{TE}}}}{{{l_{TE}}}}}}{{\mathop \sum \nolimits_{s \in T} \frac{{{T_s}}}{{{l_s}}}}}\ \end{equation*}

After the total counts (unique and multi-mapped) of each TE are re-calculated, multi-mapped reads can be re-assigned in subsequent iterations of expectation (assigning multi-mapped read fractions to TEs) and maximization (summation of unique and multi-mapped fraction counts). These iterations can be repeated until a given iteration number set by the user or until the TE counts converge (‘auto’, when all of the TEs with ≥10 counts change by <1%).

TEs with few uniquely aligning reads may be prone to misrepresentation. Users who want to identify TEs that are more likely to be false positives can examine a ‘score’ value provided in the **Count** output. The score is defined as $\frac{{{C_{TE}}}}{{{R_{TE}}}}$ × 100, where ${R_{TE}}$ represents the number of all reads aligned to the locus (unique and multi-mapping), and ${C_{TE}}$ represents the final read count from SQuIRE. A low score indicates that relatively more reads assigned to the TE are potentially derived from other loci, while a high score conveys greater certainty in the read count.

An example of **Count** output is provided in Supplementary Table S1. Further details of the **Count** algorithm are in Supplemental Methods.

### RNA-seq simulation

To evaluate SQuIRE with known TE expression levels, we tested SQuIRE with simulated RNA-seq data. We randomly selected 100 000 TEs from the GRCh38/hg38 (hg38) Repeatmasker annotation downloaded by **Fetch**. We limited our list of potential TEs to those included in TEtranscripts (41) and RepEnrich (40) to enable comparisons between these different programs. Using the selected TE coordinates we generated a BED file using **Clean** and obtained FASTA sequences using **Seek**. To mimic intrinsically regulated expression which can be more difficult to detect, we did not include flanking sequence in the TE coordinates for simulation. From these TE sequences, we used the Polyester package from Bioconductor (R version 3.4.1; (54)) to simulate 100 bp, paired-end, stranded RNA-seq reads with normally distributed fragment lengths around a mean of 250 bp. We simulated a uniformly distributed sequencing error rate of 0.5%. TEs were simulated with a mean read coverage of 20×, with 250 TEs deviating from that mean between 2- and 100-fold.

### HEK293T cell culture, transfection and sequencing

To evaluate SQuIRE with induced TE expression, we transfected a LINE-1 (L1, L1RP) expressing plasmid into a cell line and used SQuIRE to evaluate L1 expression. LINE expression constructs were cloned into the pCEP4 backbone (Thermo Fisher Scientific, Waltham, MA) modified to confer puromycin resistance. Plasmids encoded either L1RP (MT302) or had no insert (55). Tet-On HEK293TLD (293T) cells (55) were grown at 37°C, 5% CO2 in DMEM with 10% Tet-Free FBS (Takara, Mountain View, CA) and passaged every 3–5 days as needed with regular tests for mycoplasma contamination. For transfection, 300 000 293T cells were plated in 2 ml volume. 24 h later, cells were transfected using a cocktail of 2 μg plasmid DNA and 6 μl Fugene HD (Promega), and puromycin was added 24 h later for a total of 3 days of selection. 500 000 cells were then plated in three wells each, and doxycycline was added 2 h later (final concentration of 1 ug/ml) to induce L1 expression. RNA was collected after 72 h of L1 expression using the Zymo Quick-RNA MiniPrep kit (Zymo Research, Tustin, CA, USA). The RNA libraries of transfected 293T cells were prepared using the Illumina TruSeq Stranded Total Library Prep Kit with Ribo-Zero Gold (San Diego, CA, USA) to provide stranded, ribosomal RNA depleted RNA. The libraries were sequenced on an Illumina HiSeq 2500, using six samples per lane across eight lanes with paired-end 100 bp reads. We generated a mean of 263 127 067 paired reads per sample. The raw sequencing data were deposited to the NCBI Genome Expression Omnibus (GEO) with accession number GSE113960.

### HEK293T cell RNA-seq analysis and *in silico* spike-in experiment

We ran SQuIRE on HEK293T cells transfected with an L1RP expression construct (DA1) and an empty vector (DA5). To incorporate the L1RP vector into the alignment, the vector sequence was added to a custom table and referenced using the ‘—extra’ option in **Map**. We used SAMtools (49) to identify reads that align to the construct (Supplementary Table S2). To test the effect of ectopic L1RP expression on the false positive rate of endogenous L1 expression estimation, we took L1RP-aligning reads from sample DA1 for *in silico* ‘spike-in’ to sample DA5. To downsample these L1RP-aligning reads, we used the SAMtools ‘-s *<INT.FRAC>* ’ option. We used values of 0.01, 1.001 and 3.0004 as inputs, resulting in expression levels of 0.43, 0.78 and 7.90 fpkm. [The integer before the decimal indicates the seed value and the number after the decimal indicates the fraction of total alignments desired for subsampling.] We used the SAMtools ‘merge’ tool to combine these L1RP-aligning reads with one lane equivalent (29.8 million reads) from the empty vector (DA5) sample.

### Mouse data

Mouse RNA-seq data were obtained from GEO with accession number GSE30352. This study included biological replicates of brain, heart, kidney, liver and testis tissue from adult C57BL/6 mice (56). The RNA-seq data was paired-end, unstranded, with 76 bp length reads.

### TE RNA-seq tool comparison

Because SQuIRE is the first to quantify TE expression at the locus level, we restricted comparisons of SQuIRE’s performance with other TE analysis software to subfamily level analyses. All pipelines were run on a server with a maximum of 128 GB memory available and 8 threads (-p setting). For SQuIRE, we used the **Fetch** tool to obtain hg38 and GRCm38/mm10 (mm10, based on the C56BL/6 strain) genome FASTA sequences and RepeatMasker annotation from UCSC and generate a STAR alignment index. We ran **Map**, **Count** and **Call** with default settings, specifying the –build, –read_length and –strandedness parameters for the simulated human and mouse datasets. For RepEnrich (40), we obtained the hg38 annotation for RepeatMasker from the RepEnrich GitHub website and mm10 RepeatMasker (57) annotation from the RepeatMasker website. We mapped the RNA-seq data using Bowtie 1 (58) according to RepEnrich's instructions. The alignments were then used for the RepEnrich software with the ‘–pairedend TRUE’ parameter for simulated human data, and ‘–pairedend FALSE’ for mouse data. For TETools, we generated rosette files for hg38 and mm10 by taking the Repeatmasker annotation from **Clean** for the first column and the repeat taxonomy for the second column (subfamily:family:superfamily). We used the BED file from **Clean** with **Seek** to obtain TE FASTA sequences for generation of a pseudogenome for TETools. TETools was run with the ‘-bowtie2’, ‘–RNApair’ and ‘–insert 250’ parameters for simulated human data and ‘-bowtie2’, ‘-insert 76’ for mouse data. For TEtranscripts, we obtained hg38 and mm10 GTF annotation from the TEtranscripts website. We aligned the data to the genome with STAR using ‘–winAnchorMultimapNmax 100’, ‘–outFilterMultimapNmax 100’ parameters for multi-mapping. We then ran TEtranscripts with the ‘–mode multi’ setting to utilize its expectation-maximization algorithm for assigning multi-reads for the resulting SAM file. Since TEtranscripts analyzes TE and gene expression together, we used refGene annotation obtained by SQuIRE **Fetch** for the required GTF file. We used the parameters ‘–format SAM’, ‘–mode multi’, ‘–stranded yes’ for simulated human data, and ‘–format SAM’, ‘–mode multi’, ‘–stranded no’ for mouse data.

### Aligner comparison

To compare aligners used by TE analysis tools on their ability to correctly identify uniquely mapping reads, we ran the aligners Bowtie1 (58), Bowtie2 (59), and STAR (47) on the simulated TE RNA-seq data described above. We set each aligner to output a maximum of two valid alignments to quickly identify uniquely aligning reads with the parameter ‘-m2’ for Bowtie 1, ‘-k2’ for Bowtie 2 and ‘–outSAMmultNmax 2’ for STAR. We also ran STAR with the parameters ‘–outFilterScoreMinOverLread 0.4 –outFilterMatchNminOverLread 0.4 –chimSegmentMin 100’ to allow for discordant alignments, which STAR excludes by default. Bowtie2 reports discordant alignments by default, while Bowtie 1 can only report paired alignments. We used BEDTools (48) to intersect the BAM outputs to RepeatMasker annotation to identify the TEs to which the aligners mapped the reads. Reads that only appeared once were labeled as ‘uniquely aligning’. We assessed whether the mapped TE matched the templating TE for the simulated read to determine if the uniquely aligning reads mapped to the correct location.

### *Drosophila* data

*Drosophila* RNA-seq data were obtained from GEO (accession number GSE85398). This study included biological replicates of transgenic *Drosophila* expressing human TDP-43 protein (hTDP43). Expression of hTDP43 was activated by a pan-neuronal enhancer (*Elav*) or a pan-glial enhancer (*Repo*) (60). RNA-seq was performed on paired-end, unstranded, total RNA libraries with 101 bp long reads. We ran SQuIRE on the resultant data using the dm6 genome assembly, with default settings specifying the –build, –read_length and –strandedness. We performed differential expression using **Call** comparing hTDP43-expressing *Drosophila* lines (*Elav/TDP43* and *Repo/TDP43*) with control (*TDP43/2U*).

The study that had previously analyzed this dataset used TEtranscripts to analyze TE RNA expression and further aggregated their subfamily level findings to the family level (46). They confirmed findings at the subfamily level by qRT-PCR, protein immunolabeling, and RNAi silencing studies based on the *Gypsy* consensus sequence (GenBank: M12927.1) (61). The RepeatMasker track includes >9500 loci and 45 different subfamilies belonging to the *Gypsy* family (57,62). To corroborate past findings of *Gypsy* expression in this dataset, we focused our analysis on the subset of *Gypsy* loci that correspond with the *Gypsy* consensus sequence used in previous studies (61,63). We used BLAT to determine all consensus sequence-aligning loci belonging to ‘Gypsy_I’ subfamily annotations in the RepeatMasker track (57,63,64). Because the subfamily-level analyses on hTDP43-mediated *Gypsy* upregulation focused on *Gypsy* coding sequences, we used SQuIRE’s output for sequences corresponding to Gypsy-I’s internal sequence (‘Gypsy_I-int’), and excluded long terminal repeat entries which do not contain open reading frames (‘Gypsy_I-LTR’).

### Statistical analysis

Differential expression analysis of gene and TE expression was performed using DESeq2 (51) via the SQuIRE **Call** tool (see Supplemental Methods). *P*-values were adjusted for multiple-comparisons with an FDR cutoff of 0.1. To determine if loci belonging to a TE subfamily was more likely to be differentially expressed in testis compared to other TE subfamily loci, a Fisher's exact test was performed. The Fisher's exact test was chosen due to the small percentage of TE loci that are expressed.

## RESULTS

### SQuIRE overview

**Figure 1.**
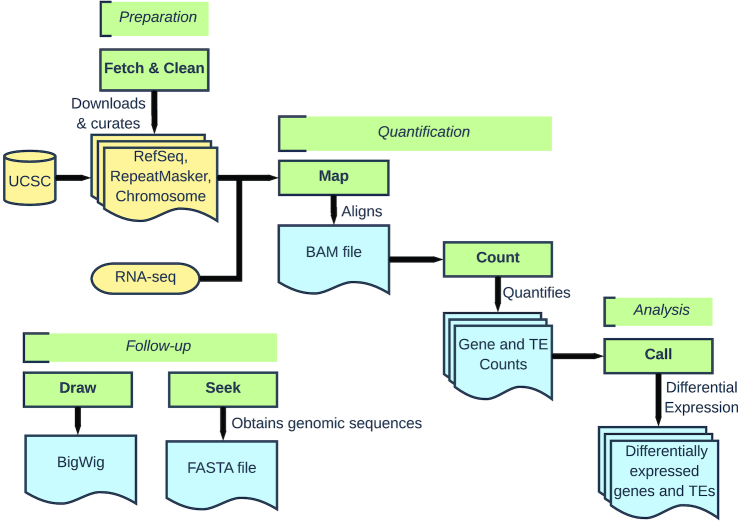
Schematic overview of the SQuIRE pipeline. Green boxes with bold text represent SQuIRE tools, with the pipeline stage (Preparation, Quantification, Analysis and Follow-up) indicated above. Yellow represents inputs to SQuIRE. Blue represents SQuIRE outputs.

SQuIRE provides a suite of tools for analyzing transposable element (TE) expression in RNA-seq data (Figure 1). SQuIRE’s tools can be organized into four stages: (i) Preparation, (ii) Quantification, (iii) Analysis and (iv) Follow-up. In the *Preparation* stage, **Fetch** downloads requisite annotation files for any species with assembled genomes available on University of California Santa Cruz (UCSC) Genome Browser (63). These annotation files include RefSeq (65) gene information in BED and GTF format, and RepeatMasker (57) TE information in a custom format. **Fetch** also creates an index for the aligner STAR (47) from chromosome FASTA files. **Clean** reformats TE annotation information from RepeatMasker into a BED file for downstream analyses. The tools in the *Preparation* stage only need to be run once per genome build. The *Quantification* stage includes the alignment step **Map** and RNA-seq quantification step **Count. Map** aligns RNA-seq data using the STAR aligner with parameters tailored to TEs that allow for multi-mapping reads and discordant alignments. It produces a BAM file. **Count** quantifies TE expression using a SQuIRE-specific algorithm that incorporates both unique and multi-mapping reads. It outputs read counts and fragments per kilobase transcript per million reads (fpkm) for each TE locus, and aggregates TE counts and fpkm for TE subfamilies into a separate file. **Count** also quantifies annotated RefSeq gene expression with the transcript assembler StringTie (50) to output annotated gene expression as fpkm in a GTF file, and as counts in a count table file. In the *Analysis* stage, **Call** performs differential expression analysis for TEs and RefSeq genes with the Bioconductor package DESeq2 (51,54). To allow users to visualize alignments to TEs of interest visualized by the Integrative Genomics Viewer (IGV) (66) or UCSC Genome Browser, the *Follow-up* stage tool **Draw** creates bedgraphs for each sample. **Seek** retrieves sequences for genomic coordinates supplied by the user in FASTA format. We describe further details of the SQuIRE pipeline in Supplemental Methods.

### Count algorithm

SQuIRE’s **Count** algorithm addresses a fundamental issue with quantifying reads mapping to TEs: shared sequence identity between TEs from the same subfamily and even superfamily. When a read fragment originating from these non-unique regions is aligned back to the genome, the read may ambiguously map to multiple loci (‘multi-mapped reads’). This is not a major problem for older elements that have acquired relatively many nucleotide substitutions, and thus give rise to primarily uniquely aligning reads (‘unique reads’). However, TEs from recent genomic insertions that have high sequence similarity to other loci may have few distinguishing nucleotides. Among elements of approximately the same age, relatively shorter TEs also have fewer sequences unique to a locus. Thus, discarding or misattributing multi-mapped reads can result in underestimation of TE expression.

Previous TE RNA-seq analysis pipelines have been able to quantify TE expression at subfamily-level resolution. The software RepEnrich (40) ‘rescued’ multi-mapping reads by re-aligning them to pseudogenome assemblies of TE loci and assigning a fraction of a read inversely proportional to the number of subfamilies to which each read aligned. These multi-mapped fractions were combined with counts of unique reads aligned to each subfamily. This approach was an advance in that it used information from multi-mapped reads. However, this method results in assigning fractions that are proportional to the number of subfamilies that share the multi-mapped read's sequence, rather than each subfamily's approximate expression level. TEtranscripts (41) expanded on this rescue method by assigning an initial fractional value inversely proportional to the number of TE loci (not subfamilies) to which each read aligned. This initial fractional value was then used in an expectation-maximization (EM) algorithm, which iteratively re-distributes fractions of a multi-mapping read among loci (*E*-step) in proportion to their relative multi-mapped read abundance estimated from a previous step (M-step). The total of multi-mapped reads and unique reads for each loci are then summed by subfamily. However, in excluding unique reads from the EM algorithm, TEtranscripts does not incorporate empirical high-confidence data to infer TE expression levels from unique TE alignments. Furthermore, in calculating the relative expression level of multi-mapped reads, TEtranscripts normalizes read counts based on annotated coordinates from RepeatMasker. This underestimates TE expression levels for transcripts shorter than the annotated genomic length. TEtranscripts then sums the unique and multi-mapping counts for each subfamily.

**Figure 2.**
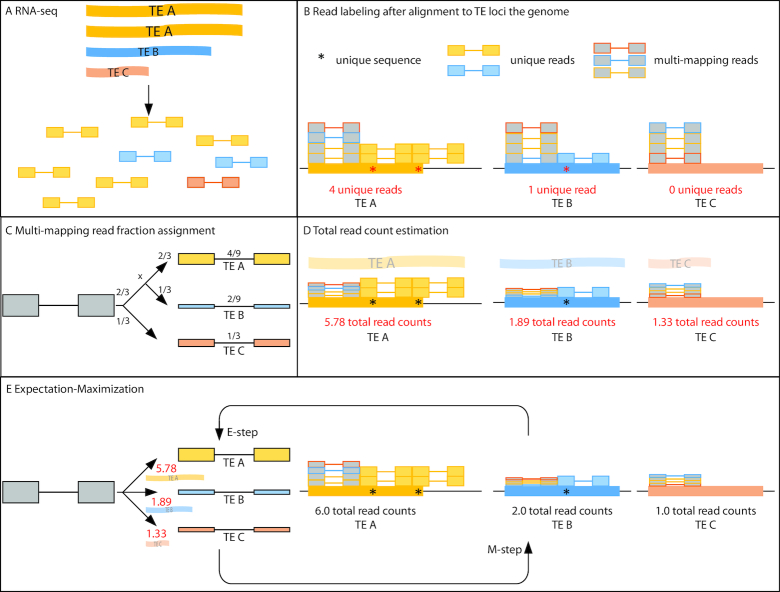
Schematic representation of the SQuIRE **Count** algorithm. This example illustrates the quantification of RNA-seq reads (paired boxes joined by a line) from three hypothetical TE transcripts (ribbons) with various expression levels and transcript lengths. (**A**) RNA transcripts are sequenced, producing paired-end reads. In this example, the lengths of TE transcripts vary such that TE A > TE B > TE C. (**B**) Identification of reads that align to TE loci in the genome. The annotated lengths of TE A, TE B, and TE C are the same; TE A is transcribed beyond the boundaries of the TE annotation, and TE C is partially transcribed such that the transcript is shorter than the annotated TE. **Count** labels reads as unique (colored boxes) or multi-mapping (grey boxes). Uniquely mapping reads map to a single TE at a unique sequence in the genome (asterisks), whereas multi-mapping reads map to similar sequence shared by the three TEs. (**C**) Next, **Count** assigns fractions of multi-mapping reads in proportion to the normalized unique read expression of each TE. TEs without uniquely aligning reads are assessed first. Because TE C has no uniquely aligning reads, it receives a fraction equal to 1/3, which is inversely proportional to the number of loci to which the multi-mapping read aligned. The remaining 2/3 fraction is apportioned to TE A and TE B relative to their unique read counts, normalized by the number of unique read positions (TE A: $\frac{{4\ unique\ reads}}{{2\ unique\ positions}} = \ 2$; TE B: $\frac{{1\ unique\ read}}{{1\ unique\ position}} = \ 1$). TE A thus receives a read fraction of $\frac{2}{3} \times \ \frac{2}{{2 + 1}} = \frac{4}{9}$, while TE B receives a read fraction of $\frac{2}{3} \times \ \frac{1}{{2 + 1}} = \frac{2}{9}$ for each of the four multi-mapping reads. (**D**) The multi-mapping fractions are summed with the unique reads to give an initial total read count estimation. E) **Count** runs an expectation-maximization loop that reassigns multi-mapping read fractions for each TE (E-step), and re-estimates total read counts (M-step) until convergence. Multi-mapping read fractions are assigned using the previous iteration’s total read counts normalized to transcript length, not the annotated length of the TE.

In order to accurately quantify TE RNA expression at locus resolution, **Count** builds on these previous methods by leveraging unique read alignments to each TE to assign fractions of multi-mapping reads (Figure 2) and then iteratively improving those assignments. First, **Count** distinguishes reads that uniquely map to particular TE loci (‘unique reads’) from reads that ambiguously map to multiple locations (‘multi-mapped reads’). Second, the count of a multi-mapped read is divided into fractions allocated to different TE loci in proportion to each TE’s unique read count, normalized to uniquely alignable length. Third, the unique reads and multi-mapped read fractions are summed and normalized to each TE’s transcribed length. Finally, the normalized total read counts are used in an expectation-maximization (EM) loop to reallocate multi-mapped read fractions. SQuIRE **Count** is the only TE RNA-seq analysis tool to output the length and strandedness of each TE transcript based on aligned read positions. The outcomes of using only unique reads, ignoring unique reads in multi-mapping read assignment, or relying on annotated TE lengths are illustrated in Supplementary Figure S1. In using the empirically derived uniquely alignable length and transcribed length to normalize TE counts, SQuIRE improves multi-mapping read assignment to allow TE RNA quantification at the locus level.

### Assessing Count accuracy in simulated data

To test the performance of SQuIRE **Count**, we simulated RNA-seq data from 100 000 randomly selected TEs from the human GRCh38/hg38 (hg38) RepeatMasker annotation. To mimic intrinsically regulated TE expression with a wide range of expression levels, TEs were simulated with read coverages ranging from 2× to 4000× and simulated counts ranging from 2 to 4588. We first evaluated accuracy by how closely SQuIRE **Count** output corresponded to the simulated read counts (i.e. % Observed/Expected). However, using this calculation is not meaningful for TEs with low simulated counts: a TE with 0 counts gives an infinite value, and a reported count of 1 for a TE with two simulated reads gives a low 50% Observed/Expected. Thus, we were primarily interested in ‘expressed’ simulated TEs, considering only the 99 567 TEs with at least 10 simulated reads. Second, we evaluated SQuIRE by how often it correctly detected simulated TE expression (i.e. true positives) or misreported unexpressed TEs (i.e. false positives).

To test how well SQuIRE performed leveraging only uniquely aligning read information, we first evaluated the % Observed/Expected of TE counts with 0 EM iterations. We found that SQuIRE accurately assigned read counts to most TEs, with a mean % Observed/Expected of 98.79% (Supplementary Figure S2). A subset of evolutionarily young subfamilies from the LINE-1 superfamily (i.e. L1PA1 or L1HS) (67), the SINE *Alu* superfamily (e.g. *Alu*Ya5, *Alu*Ya8, *Alu*Yb8, *Alu*Yb9) (68), as well as composite SVA (SINE-variable number tandem repeat (VNTR)-*Alu*) elements (69) remain retrotranspositionally active in the human genome and generate new polymorphic insertions (37,38). These new insertions share sequence from their parent copy. We expected that SQuIRE’s accuracy would be lower for younger TEs with less uniquely alignable sequence. Indeed, SQuIRE was less accurate for elements with less than 10% divergence (mean of 77.35% Observed/Expected). The most frequently retrotranspositionally active TEs (i.e. *Alu*Ya5, *Alu*Ya8, *Alu*Yb8, *Alu*Yb9 and L1HS) had counts ranging from 48% to 70% Observed/Expected, with a range of 79–92% Observed/Expected at the subfamily level (Supplementary Table S3). This illustrates that even without the EM-algorithm, SQuIRE can distinguish expression from highly homologous TEs at the subfamily level.

**Figure 3.**
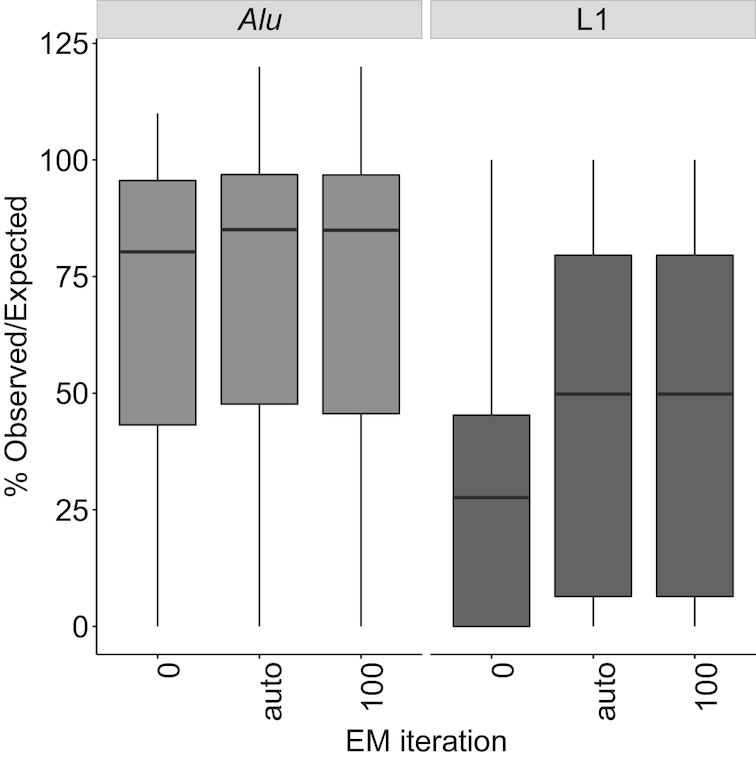
EM algorithm improves % Observed/Expected for young TEs. Running EM iterations improves the % Observed/Expected for SQuIRE **Count** for the frequently retrotranspositionally active *Alu* (*Alu*Ya5, *Alu*Ya8, *Alu*Yb8, *Alu*Yb9) and L1 (L1HS) subfamilies compared to no EM iterations (*i* = 0), and does not degrade with increasing iterations (*i* = 100). By default (*i* = ‘auto’), SQuIRE **Count** continues the EM-algorithm until each TE with >10 reported read counts changes by <1%.

Given the low recovery of simulated counts for younger elements when relying solely on uniquely aligning reads, we next evaluated how much adding the EM-algorithm improved **Count's** performance. We anticipated that the counts for most TEs would not change, but that younger elements with less divergence would have improved recovery of simulated reads. Indeed, the overall % Observed/Expected counts of TE loci increased only slightly by 0.14% to a total of 98.93%. However, the change in % Observed/Expected of TEs was much greater for the most homologous active elements, improving by 20.47% for young *Alu* elements and by 21.1% for L1HS loci (Figure 3). At the subfamily level, the % Observed/Expected of active TEs was improved by 8.1% for young *Alu* elements and by 2.2% for L1HS (Supplementary Table S3). Using updated transcript information in the EM-algorithm is thus particularly useful for TE biologists interested in younger elements that have previously been problematic to quantify by RNA-seq.

We also wanted to evaluate SQuIRE’s ability to distinguish whether a TE is expressed or not expressed. To examine how well **Count** detected expressed TEs, we calculated the true positive rate (TPR) as the percentage of TEs with at least 10 simulated reads that SQuIRE also reported to have ≥10 counts. Conversely, we evaluated how often SQuIRE falsely reports TE expression by calculating the positive predictive value (PPV) as the percentage of TEs with ≥10 reported counts that were in fact simulated to have ≥10 reads. The true negative rate, or how often SQuIRE correctly reports that a TE is *not* expressed, is less informative for evaluating TE estimation accuracy because the number of TEs in the hg38 genome is so high (>4 million TEs) that the true negative value would outweigh the false positive value (70). Overall, SQuIRE had both a high TPR of 98.5% and high PPV of 99.4%. These values were lower for frequently retrotranspositionally active *Alu* elements (TPR = 68.75–83.33%, PPV = 64.29–100%) and L1HS elements (TPR = 100%, PPV = 62.86%) using only unique reads for TE expression estimation (Supplementary Table S4). However, using the EM algorithm improved the TPR for *Alu* loci (TPR = 85.22–100%) by reducing false negative reports, and improved the PPV for L1HS loci (PPV = 78.57%) by reducing false positives. The inclusion of false positives in analysis can be further reduced by imposing a score threshold. A low score indicates that multi-mapping reads contribute significantly to the read count. When we plotted the TPR and PPV using various score thresholds, we found that using a score threshold of at least 50% maximized the combination of TPR and PPV for TEs in the hg38 genome build (Supplementary Figure S3).

### LINE-1 detection with Count *in vitro*

To evaluate how **Count** handles expression from young TEs, we transfected HEK293T cells with a plasmid containing an L1HS known as L1RP (71,72). Like endogenous TEs, RNA expression from the L1RP plasmid includes unique 5′ and 3′ sequence flanking the L1HS sequence. SQuIRE readily detected the ectopic transcript, which was 686-fold more highly expressed than the highest L1HS locus in control cells (301.97 fpkm versus 0.44 fpkm).

To evaluate whether **Count** would favor the L1RP over endogenous loci due to its unique flanking sequence, we ‘spiked in’ L1RP plasmid-aligning reads to the RNA-seq alignment files of HEK293T cells transfected with empty plasmid. We used randomly downsampled reads at levels approximating 1×, 2× and 20× the highest expressing endogenous locus level. We then looked at the read counts of endogenous L1HS loci before and after ‘spike-in’. Before ‘spike-in’, 22 L1HS loci were detected with >10 counts, 5 of which initiated transcription at the L1HS promoter (Supplementary Figure S4). At all simulated L1RP expression levels, there were no L1HS loci with decreased read counts after L1RP spike in. This suggests that **Count** appropriately normalizes for each transcript's uniquely alignable sequence.

**Figure 4.**
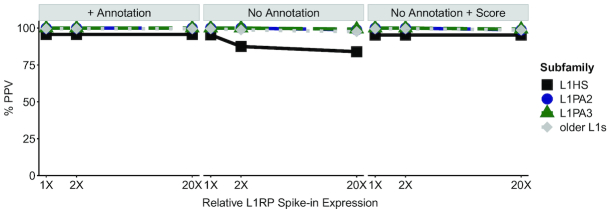
SQuIRE has high Positive Predictive Value for L1 loci. Positive predictive value (PPV) of L1 loci expression in HEK293T cells when ‘spiking-in’ L1RP-aligning reads. The three youngest subfamilies—L1HS (also known as L1PA1), L1PA2, L1PA3 are shown here separately, while older L1 subfamilies are analyzed together. False positive expression is implicated if a locus that previously had <10 reads has ≥10 reads after ‘spike-in’, while loci with true positive expression had ≥10 reads both before and after ‘spike-in’. % PPV is the percentage of loci with true positive loci relative to the total number of loci with ≥10 SQuIRE read counts. The PPV is robust for increasing ‘spike-ins’ equivalent to 1×, 2× and 20× the RNA levels of the most highly expressed endogenous full-length L1HS locus. ‘Spike-in’ reduces PPV for L1HS modestly in a dose dependent manner (center panel), and this can be mitigated by adding an annotation for the ‘spiked’ L1HS (left panel) or imposing a score threshold >50 (right panel).

Conversely, because L1RP has 99.9% sequence identity to the consensus sequence of all L1HS copies, we wanted to assess if ‘spiking-in’ multi-mapping L1RP reads would result in misattributed reads to low-expressing L1 loci. Of the L1RP-aligning reads that were spiked in, only 46–50% contributed to the total read count of the L1RP locus. To assess if the remaining reads affect estimates of expression at other L1 loci, we calculated the number of false positive L1 loci that became ‘expressed’ with >10 counts after the *in silico* ‘spike-in’ and how this affected the PPV. We focused on the three youngest L1 subfamilies that share the greatest homology with the L1RP sequence (i.e. L1HS or L1PA1, L1PA2 and L1PA3) (73–75) and compared their false positive rates to older L1 loci (Figure 4). We found that ‘spiking-in’ L1RP-derived reads only resulted in 1 false positive L1HS locus for a PPV of 95.6%, while the older subfamilies had PPVs of 99.6–100% (Figure 4). The PPVs did not change with increasing L1RP expression. Thus, there is negligible misattribution of reads.

Because some L1HS loci remain retrotranspositionally active, they can generate insertions that are polymorphic or novel compared to the reference human RepeatMasker annotation. To assess how expression from a novel L1HS locus can impact the quantitation of reference L1 loci, we removed the L1RP annotation from the **Map** alignment and re-ran **Count** on the ‘spiked-in’ data. We found that **Count** was still able to detect 21 out of 22 L1HS loci (95.4%). There was an increase in false positively reported loci, decreasing the PPV from 95.4% to 84.0% with increasing L1RP expression from 1× to 20× the highest expressing endogenous L1HS locus (Figure 4). The PPV remained high for the older L1 subfamilies, ranging from 97.5% to 100%. When we set a score threshold of >50, the PPV of L1HS returned to 95.2% for all ‘spike-in’ levels, with only 1 false positive L1HS locus reported. Using a score of >50 did not drastically reduce the detection of expressed L1HS loci, with 20 of 22 (90.9%) still meeting the threshold. Thus, while estimated expression of young L1HS elements may be affected by transcription from polymorphic insertions, accuracy can be improved by adding TE annotation or using a score threshold.

### Comparison to other software

Currently published TE analysis software include RepEnrich, TEtranscripts and TETools (40–42). TEtranscripts has previously illustrated the improvements of using TE-targeted software for quantifying TE expression compared to conventional pipelines (41). Because none of these programs is capable of reporting TE locus expression, we performed comparisons with SQuIRE with aggregated subfamily estimates. We used the simulated hg38 TE data described above to compare the recovery of simulated reads to the correct subfamily among TE quantification software (i.e., % Observed/Expected). For mapping, we ran each software's recommended aligner: STAR (used by SQuIRE and TEtranscripts), Bowtie 2 (used by TETools), and Bowtie 1 (used by RepEnrich). We found that SQuIRE (99.86 ± 1.46%), TETools (100.14 ± 2.21%), and TEtranscripts (95.89 ± 16.41%) had comparable % Observed/Expected rates (Supplementary Figure S5). In contrast, RepEnrich (108.77 ± 40.67%) was less accurate in terms of % Observed/Expected. This is likely attributable to RepEnrich's recommended settings for Bowtie 1, which discards discordant reads and limits the number of attempts to align both paired-end mates to repetitive regions. To support this, we compared how often each aligner mapped a uniquely aligning simulated read to the correct location. We indeed found that Bowtie 1 failed to report unique reads more often in a paired-end library compared to single-end (Supplementary Table S5).

To compare SQuIRE to other TE analysis tools with biological data, we ran each pipeline on publicly available adult C57Bl/6 mouse tissue RNA-seq data (56) using GRCm38/mm10 (mm10) TE annotation. We compared the expression of subfamilies in testis compared to pooled data from brain, heart, kidney and liver tissues. To independently evaluate the fold-changes of TE RNA between testis and somatic tissues, we also used our previously published adult C57Bl/6 mouse Nanostring results (85). Unlike RNA-seq analysis, which infers transcript levels by counting reads, Nanostring uses uniquely mapping probes to capture and count RNA molecules. It thus provides an orthogonal, alignment-independent approach with which to compare TE RNA-software pipelines. We compared the Nanostring log_2_ fold changes (log_2_FC) of TE subfamily expression in testis and pooled somatic tissue to the log_2_FC values found by SQuIRE, RepEnrich, TEtranscripts, and TETools (Supplementary Figure S6). Because the Nanostring probes were designed against TE consensus sequences, we do not expect exact correspondence with the RNA-seq analysis tools. We observe elements for which all TE RNA-seq tools report results opposing the Nanostring result (MMVL30, IAPLTR1a_Mm, RLTR13A1). Thus in addition to comparing each pipeline with Nanostring, we also evaluated when a result deviated from the other TE RNA-seq analysis pipelines. RepEnrich failed to detect differential expression of the L1_mus_musculus subfamily (L1_Mm), and reported a direction of log_2_FC for the MMETn subfamily that opposed Nanostring results. TEtranscripts similarly failed to detect differential expression of MMERVK10D3 subfamily that Nanostring and the other pipelines reported, and reported different log_2_FC from Nanostring, SQuIRE, and TETools for L1Mm. TETools deviated from Nanostring and the other RNA-seq pipelines for the MERVL subfamily, reporting decreased expression in testis while the other methods reported upregulation. Thus, SQuIRE is the only RNA-seq pipeline producing results that corresponded with at least two other methods.

### Locus-level TE expression analysis

**Figure 5.**
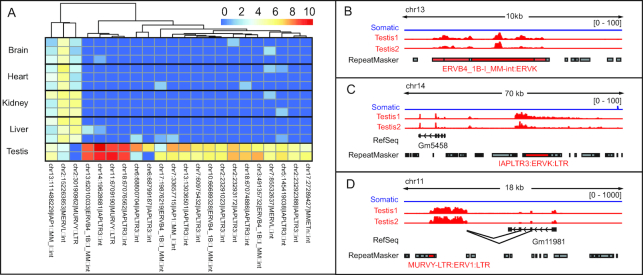
Differentially expressed TEs are transcribed as part of different transcript types. (**A**) The X-axis represents replicates of somatic and testis tissue samples from adult C57Bl/6 mouse. The Y-axis represents differentially expressed TE loci. The heatmap colors represent the log_2_ of total read counts +1 for each TE locus. (**B**–**D**) Examples of intergenic TE loci differentially expressed in testis compared to somatic tissues. Tracks from brain, heart, kidney and liver replicates were collapsed into a single track. The scales of count expression are shown in brackets. The RefSeq track represents annotated genes. The RepeatMasker track represents transposable elements annotated in the reference genome. Transposable elements colored in red belong to the subfamily indicated; dark red indicates that that RepeatMasker entry meets significant differential expression thresholds (log_2_FC > 2, padj < 0.05).

With SQuIRE, we can closely examine the mouse RNA-seq data at the locus level. For the 16 subfamilies analyzed by Nanostring and the TE analysis tools, using SQuIRE we found that the reported subfamily-level expression was due to expression from fewer than 7% of each subfamily's loci (Supplementary Figure S7). While most subfamilies studied by Nanostring have only 1–4 significantly differentially expressed loci (log_2_FC > 1, padj < 0.05), the IAPLTR3 subfamily has 11 loci that are all differentially expressed in testis compared to somatic tissues (Figure 5A). To test whether this was an enrichment relative to the representation of IAPLTR3 in the mouse genome, we performed a Fisher's exact test and found that IAPLTR3 loci were 10-fold more likely than expected to be differentially expressed in testis (OR: 10.56, 95% CI: 5.25–18.97, *P*-value < 1.61e–08). ERVB4-1B, another LTR retrotransposon that exhibited high fold change by Nanostring, was not similarly enriched among differentially expressed TE loci. In addition to a more careful analysis of which loci are transcribed, SQuIRE enables a closer look at TE transcript structure. In examining the TE loci with the greatest differential expression in testis, we found that the transcription of the ERVB4-1B locus on chr13 did not extend beyond annotations for that element (Figure 5B), suggesting intrinsically regulated expression. On the other hand, the IAPLTR3 loci on chr14 (Figure 5C) and chr18 are part of longer transcripts that initiate outside of the annotated TE. Altogether, this suggests while a subset of TEs may be regulated by shared TE sequence, most differential expression of TEs is locus-specific with varying transcript structures, a finding that was not evident until analysis at the locus level using SQuIRE.

To further investigate the interplay between genomic context and TE subfamily, we identified the closest genes to differentially expressed TE loci. We found a cluster of three loci exhibiting broad expression across somatic tissues from the IAP1, MERVL, and MURVY LTR retrotransposon subfamilies. When we examined the genomic context of these 3 loci, we found that all were located within genes with known broad tissue expression (*Gpbp1, Csnk2a1, Kyat1*, respectively) (86), with examples shown in Supplementary Figure S8. Another locus from the MURVY subfamily is in a cluster of TEs exhibiting high testis-restricted expression. In examining the transcript overlapping the MURVY locus, we see that the transcript initiates outside of the locus and find that the transcript is an alternative splicing isoform with splice donors from the third and fourth exons of a gene ∼5 kb away (Figure 5D). The gene, *Gm11981*, is a long noncoding RNA (lncRNA) known to exhibit testis-restricted expression (86). The different MURVY-containing transcript types illustrate how TE transcription can vary across loci from the same subfamily. Altogether, these findings would be lost without the use of SQuIRE to analyze TE transcription at the locus level.

### Locus-level analysis of TE expression in transgenic hTDP43 *Drosophila* model

To further illustrate the importance of locus-level analysis of TE expression with SQuIRE, we applied the SQuIRE pipeline to a *Drosophila melanogaster* model of amyotrophic lateral sclerosis (ALS) (46,60). Almost all (97%) of ALS patients develop cytoplasmic inclusions of the TDP-43 protein (87). TDP-43 is a DNA- and RNA-binding protein that is involved in the regulation of RNA splicing, microRNA biogenesis, transcriptional repression, and cell stress responses (87–90). Toxicity from cellular aggregation of TDP-43 has been previously shown to impact both neurons and glial cells (89,91–93). This *Drosophila* model conditionally expresses human TDP43 protein (hTDP43) in a Gal4/UAS system activated by Gal4 drivers (60). Overexpression of hTDP43 in neurons (*Elav/TDP43*) or glia (*Repo/TDP43*) replicates clinical and pathological features of ALS (46,60,91,92). This dataset had been previously analyzed using TEtranscripts at the family level, which found upregulation of several TE families with hTDP43 expression as compared to control samples with no hTDP43 expression (*TDP43/2U*) (46). Pan-glial expressing hTDP43 *Drosophila* brains particularly feature increased TE expression, with upregulation of 23 among the 29 differentially expressed families. Among the 23 TE families increased in *Repo/TDP43 Drosophila, Gypsy* was of special interest (59)*. Gypsy* is a retrotranspositionally active endogenous retrovirus that has previously been reported to generate new insertions in aging *Drosophila* brain (94). Pan-glial, but not pan-neuronal hTDP43-mediated *Gypsy* expression and its associated toxicity was confirmed by subfamily-level qRT-PCR, protein immunolabeling and RNAi silencing. Their findings suggested that upregulation of *Gypsy* in glial cells contribute to the decreased lifespan of *Repo/TDP43 Drosophila*.

**Figure 6.**
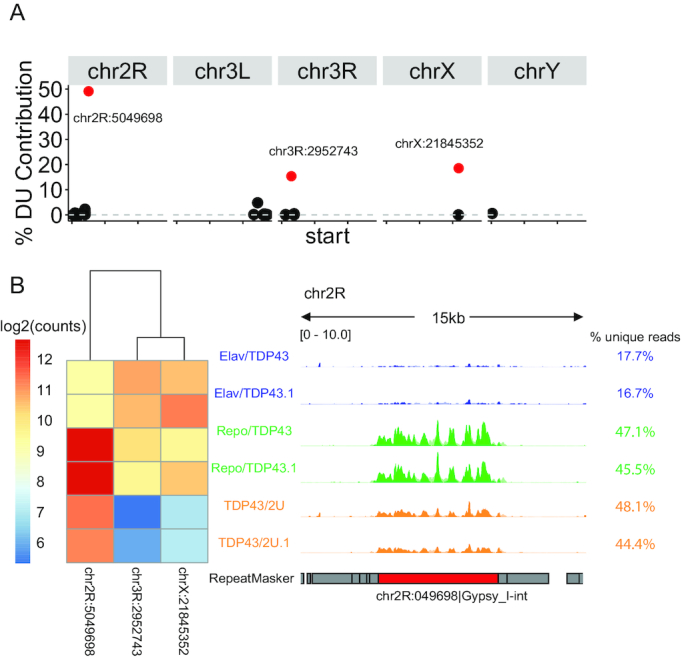
TDP43 upregulates the *Gypsy* endogenous retrovirus at few specific loci. (**A**) Percent contribution of differential upregulation (% DU contribution) for each *Gypsy* loci (circle) with positive finite log2FC in *Repo/TDP43* samples compared to controls. X-axis: chromosome position, each tick representing 10Mb. Y-axis: % DU contribution as log2FC scaled by locus expression levels as a percentage of total scaled log_2_FC for all loci. The loci colored in red (located on chr2R, chr3R, and chrX), have the greatest contribution to *Gypsy* differential expression. (**B**) *Gypsy* expression patterns for pan-neuronal (*Elav/TDP43*), pan-glial (*Repo/TDP43*), and no (*TDP43/2U*) TDP43 samples (performed in duplicate). Left: Heatmap of log_2_ transformed counts for each *Gypsy* locus with > 10% DU contribution. Right: *Gypsy* expression track for the chr2R locus, which had the greatest % DU contribution. Dark colors represent unique alignments, while pale colors represent multi-mapped alignments. The heights of multi-mapped alignments are inversely proportional to the number of loci to which the reads aligned. To the right of each track are the % of all alignments to the 2R locus that are uniquely aligned.

We investigated how *Gypsy* upregulation by hTDP43 expression reflected differential expression of individual *Gypsy* copies at the locus level. To corroborate previous subfamily-level findings, we excluded 44 out of 45 *Gypsy* subfamilies that did not correspond to the sequence used for *Gypsy* qPCR, antibody, and RNAi design (61,95,96). Because we were focused on examining the previously reported pan-glial hTDP43 mediated Gypsy upregulation, we excluded 46 *Gypsy* loci (of the 102) that exhibited negative log_2_ fold changes in *Repo/TDP43* samples compared to controls or had infinite fold changes due to low expression across samples. To gauge the percentage that each remaining locus contributed to *Gypsy* differential upregulation (% DU contribution), we scaled the log_2_FC values by the total read count across all samples for each locus and divided by the sum of all scaled log_2_FC values. We identified 3 loci with the greatest (>10%) contribution to *Gypsy* differential upregulation in *Repo/TDP43* samples (Figure 6A). All three loci were significantly differentially expressed in a pairwise comparison between *Repo/TDP43* and control samples (chr2R:log_2_FC 1.31 padj 6.23e–24, chr3R: log_2_FC 2.58, padj 1.027e–13, chrX: log_2_FC 3.76, padj 1.43e–37). In examining the normalized counts at these loci, we were surprised to find that two loci located on chromosomes 3R and X exhibited even greater differential upregulation with pan-neuronal hTDP43 expression than with pan-glial hTDP43 expression, a result that was obscured by previous subfamily-level observation (Figure 6B, left). Only the *Gypsy* locus on chromosome 2R exhibited greater glial upregulation in *Repo/TDP43* samples as previously reported. However, because the 2R *Gypsy* locus was expressed at higher levels, it made a greater contribution (49.1%) to the total measured *Gypsy* upregulation observed by TEtranscripts. Although the *Gypsy* element is still retrotranspositionally active and generating new insertions in the *Drosophila* genome, the alignments to the 2R locus include unique reads that are unlikely to come from a non-reference *Gypsy* element (Figure 6B, right). Thus, the expression pattern previously observed at the subfamily level is largely explained by a single locus. Our results demonstrate that subfamily-level analyses miss the locus-specific nature of *Gypsy* upregulation by hTDP43.

### Benchmarking for SQuIRE’s memory usage and running time

**Figure 7.**
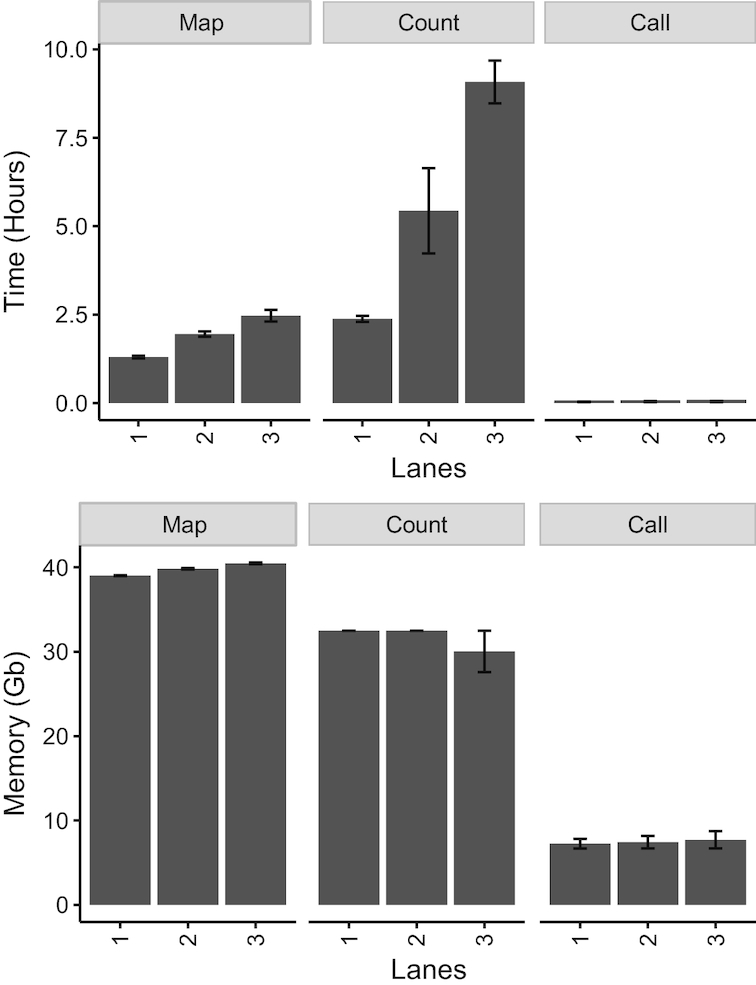
SQuIRE Benchmarking. Usage data for the main modules of SQuIRE. Time (h) and memory (Gb) for SQuIRE **Count, Map** and **Call**. Mean library sizes for RNA seq data were one lane = 32 912 528 reads, two lanes = 65 573 850 reads, three lanes = 98 757 439 reads.

To benchmark SQuIRE’s memory usage and running time for RNA-seq data of different sequencing depths, we subset the high-depth (mean 263 million reads across eight lanes) HEK293T cell line RNA-seq data into 1, 2 and 3-lane libraries with a mean sequencing depth of 32, 65 and 98 million reads. We evaluated the speed and memory performance of each *Quantification* and *Analysis* stage tool for each sequencing depth (Figure 7) using eight parallel threads and 64 Gb of available memory. We found that sequencing depth had the greatest effect on **Count**, taking 8.6 h to complete the 3-lane library compared to 2.4 h for the one lane library. The other tools took much less time and were less affected by sequencing depth. **Map** took 1–2 h for the different libraries. **Call** running time was also independent of library size, but it was greater when including all TE counts (10 min) compared to subfamily counts (2 min). We found that the memory usage of each tool was largely independent of sequencing depth, taking between 39–40 Gb of Memory for **Map**, 30–32 Gb for **Count**, and 7–8 Gb for **Call**.

### Implementation

Our efforts at making SQuIRE easy to use has resulted in multiple features in addition to its ability to provide locus-level TE quantification (Table 1). To set up SQuIRE involves a simple installation process in which the user can copy and paste lines of code, which includes instructions for setting up prerequisite software. In addition, SQuIRE is the only program that downloads reference annotation for assembled genomes available on UCSC, allowing it to be easily adaptable to a variety of species. For genomes from non-model organisms or organism strains with high divergence from the reference annotation, SQuIRE can also use RepeatMasker software output for even wider compatibility. To ensure that the pipeline is streamlined and that the outputs are reproducible, SQuIRE also implements alignment and differential expression for the user. In making SQuIRE as user-friendly as possible, we intend to improve reproducibility of bioinformatics analyses in the TE field.

**Table 1.** Feature comparison of RNA-seq Analysis tools for TEs

	SQuIRE	RepEnrich	TEtranscripts	TETools
Provides Locus-level TE RNA quantification	**YES**	–	–	–
Provides TE transcript information	**YES**	–	–	–
Copy-and-paste installation	**YES**	–	–	–
Provides prerequisite annotation files for any species	**YES**	**–**	**–**	–
Can incorporate non-reference TEs	**YES**	**–**	**–**	**YES**
Performs alignment	**YES** – uses STAR	Recommends Bowtie 1	Recommends STAR	**YES** – uses Bowtie 1 or Bowtie 2
Uses genome for alignment	**YES**	**YES** - Genome + TE pseudogenome	**YES**	**–**
Provides gene expression quantification	**YES**	–	**YES**	**–**
Performs differential expression	**YES**	–	**YES**	**YES**

## DISCUSSION

We have developed SQuIRE to characterize TE expression using RNA-seq data. TEs are highly repeated in the genome, which can pose challenges for mapping reads unambiguously to specific transcribed loci. SQuIRE is the first RNA-seq analysis software that provides locus-specific TE expression quantification while also outputting subfamily-level expression estimates (Table 1). Our approach incorporates unambiguously mapping reads as well as ambiguously mapping reads, optimally adjudicating alignments of the latter using an EM algorithm. SQuIRE additionally provides empiric information on the structure of each TE transcript rather than relying on TE annotations, recognizing that TE transcripts can be shorter or longer, and sense or antisense compared to the genomic TE. We have shown that SQuIRE correctly attributes a high percentage of reads originating from TEs using simulated data. For older, retrotranspositionally inactive genomic repeats, SQuIRE very accurately assesses expression. These older elements represent the vast majority of TE loci in the human genome (>96.7%).

Although the detection of reads is lower for frequently retrotranspositionally active, less divergent TEs (e.g. *Alu*Ya5, *Alu*Ya8, *Alu*Yb8, *Alu*Yb9, L1HS), we found that implementation of the EM algorithm (41,97) improves accuracy and lowers both false positive and false negative calls of whether a TE locus is expressed. This finding also holds in biological settings, where SQuIRE is able to correctly detect L1HS expression when we express an ectopic sequence. It maintains a low false positive rate of misattributing these reads to endogenous L1HS loci. The ongoing activity of TEs also results in a significant number of mobile element insertion variants (MEI) (37,83,98). Numerous commonly occurring structural variants owed to retrotransposition are missing in reference genome assemblies. Although these variants are not included in the default SQuIRE pipeline, SQuIRE provides users with two options to query transcription of these repeats. First, SQuIRE can detect transcription of polymorphic elements at the subfamily level. Secondly, SQuIRE can directly use sequences of known, non-reference TE insertion polymorphisms to detect locus-specific expression when these are supplied as a supplement to the reference build. For example, in the human genome, L1HS element sites and sequences can be obtained by targeted TE insertion mapping (76–79) or whole genome sequencing (80–82,84). Polymorphic TE insertions have been reported to databases such as euL1db (99), dbRIP (100) and by large studies like the 1000 Genomes Project (83). Using SQuIRE to detect expression of user-provided, non-reference TE sequences at these loci may be a useful feature for understanding functional consequences of these insertion variants (101). This confirms that SQuIRE can detect the expression of TEs in the reference genome that have in the past been problematic for global TE RNA expression analysis. For all TEs, SQuIRE provides the convenience of differential TE expression analysis with both locus-specific and subfamily-aggregated outputs.

The SQuIRE algorithm builds on strategies used by previous TE analysis software (40–42,102,103). SQuIRE rescues multi-mapping reads aligned to TEs, which improves upon pipelines that only utilize uniquely aligning reads. A similar rescue strategy had been previously applied to multi-mapping CAGE-seq tags (102). SQuIRE reduces bias against TEs without unique sequence by first normalizing to uniquely alignable length. Although expectation-maximization algorithms have been previously used in TEtranscripts and the RNA-seq quantification software RSEM, SQuIRE differs from these by normalizing to transcribed TE length rather than annotated length. Without transcribed length information, repeated iterations can perpetuate a ‘poor gets poorer’ cycle in which an underestimation of partially transcribed TE expression levels worsens with each iteration. Furthermore, because TEs can be transcribed beyond their annotation, TE-analysis strategies that align to the transcriptome instead of the genome (103) miss potential unique read alignments to flanking sequence and fail to capture the genomic context of TE expression. Here, we show that these additional features in SQuIRE’s **Count** algorithm improve on the accuracy of TE quantification, as assessed using both simulated reads and orthogonal approaches to measure log_2_ fold changes in mouse tissue comparisons. Our findings suggest that important biologic insights can be gained by examining TE transcription at the locus level.

To date, locus-specific studies of TE expression and activity have mostly focused on identifying transcriptionally and retrotranspositionally active L1s in the human genome (43–45,98,104–106). While these targeted methods can enrich for expressed TEs, they require tailored approaches for sequencing library preparation and do not yet have accompanying software. SQuIRE provides a complementary tool that supplies a software package applicable to a broad array of conventional RNA-seq datasets. These focused studies have shown that rare, individual loci, widely distributed in the genome generate RNA transcripts. In applying SQuIRE to study locus-specific TE expression genome-wide in mouse tissues and a *Drosophila* disease model, we can see that this paradigm is not unique to L1s or humans. It seems a limited subset of TE loci are transcribed with complex patterns of tissue-specific expression. Furthermore, we found that the tissue expression patterns of TE loci reflect a variety of transcriptome contexts: broadly expressed mRNA transcripts, tissue-specific lncRNAs, and *intrinsically* regulated TE transcripts. How these TEs may affect gene regulation or biological processes remain open questions. Genome-wide analyses of TEs have indicated roles for *cis*-acting elements on transcriptional regulation (3,7,107,108), transcript splicing, and RNA function (17,109–111). By providing locus-level TE transcript estimations, we expect SQuIRE will enable studies that dissect the impacts of TE expression.

## DATA AVAILABILITY

SQuIRE is freely available for download through https://github.com/wyang17/SQuIRE under the GPL-3 license. The raw sequencing data and SQuIRE Count output for HEK293T cell transfection were deposited to the NCBI Genome Expression Omnibus with accession number GSE113960.

## Supplementary Material

Supplementary Data

## References

[1] Lander E.S., Linton L.M., Birren B., Nusbaum C., Zody M.C., Baldwin J., Devon K., Dewar K., Doyle M., FitzHugh W. et al. Initial sequencing and analysis of the human genome. Nature. 2001; 409:860–921. 11237011 10.1038/35057062

[2] Kazazian H.H. Mobile elements: drivers of genome evolution. Science. 2004; 303:1626–1632. 15016989 10.1126/science.1089670

[3] Faulkner G.J., Kimura Y., Daub C.O., Wani S., Plessy C., Irvine K.M., Schroder K., Cloonan N., Steptoe A.L., Lassmann T. et al. The regulated retrotransposon transcriptome of mammalian cells. Nat. Genet. 2009; 41:563–571. 19377475 10.1038/ng.368

[4] Medstrand P., van de Lagemaat L.N., Mager D.L. Retroelement distributions in the human genome: variations associated with age and proximity to genes. Genome Res. 2002; 12:1483–1495. 12368240 10.1101/gr.388902 PMC187529

[5] Jordan I.K., Rogozin I.B., Glazko G.V., Koonin E. V Origin of a substantial fraction of human regulatory sequences from transposable elements. Trends Genet. 2003; 19:68–72. 12547512 10.1016/s0168-9525(02)00006-9

[6] de Souza F.S.J., Franchini L.F., Rubinstein M. Exaptation of transposable elements into novel cis-regulatory elements: is the evidence always strong. Mol. Biol. Evol. 2013; 30:1239–1251. 23486611 10.1093/molbev/mst045 PMC3649676

[7] Xie M., Hong C., Zhang B., Lowdon R.F., Xing X., Li D., Zhou X., Lee H.J., Maire C.L., Ligon K.L. et al. DNA hypomethylation within specific transposable element families associates with tissue-specific enhancer landscape. Nat. Genet. 2013; 45:836–841. 23708189 10.1038/ng.2649 PMC3695047

[8] Huda A., Tyagi E., Mariño-Ramírez L., Bowen N.J., Jjingo D., Jordan I.K. Prediction of transposable element derived enhancers using chromatin modification profiles. PLoS One. 2011; 6:e27513. 22087331 10.1371/journal.pone.0027513 PMC3210180

[9] Feschotte C. Transposable elements and the evolution of regulatory networks. Nat. Rev. Genet. 2008; 9:397–405. 18368054 10.1038/nrg2337 PMC2596197

[10] Chuong E.B., Rumi M.A.K., Soares M.J., Baker J.C. Endogenous retroviruses function as species-specific enhancer elements in the placenta. Nat. Genet. 2013; 45:325–329. 23396136 10.1038/ng.2553 PMC3789077

[11] Chuong E.B., Elde N.C., Feschotte C. Regulatory evolution of innate immunity through co-option of endogenous retroviruses. Science. 2016; 351:1083–1087. 26941318 10.1126/science.aad5497 PMC4887275

[12] Trizzino M., Park Y., Holsbach-Beltrame M., Aracena K., Mika K., Caliskan M., Perry G.H., Lynch V.J., Brown C.D. Transposable elements are the primary source of novelty in primate gene regulation. Genome Res. 2017; 27:1623–1633. 28855262 10.1101/gr.218149.116 PMC5630026

[13] Chuong E.B., Elde N.C., Feschotte C. Regulatory activities of transposable elements: from conflicts to benefits. Nat. Rev. Genet. 2017; 18:71–86. 27867194 10.1038/nrg.2016.139 PMC5498291

[14] Wang T., Zeng J., Lowe C.B., Sellers R.G., Salama S.R., Yang M., Burgess S.M., Brachmann R.K., Haussler D. Species-specific endogenous retroviruses shape the transcriptional network of the human tumor suppressor protein p53. Proc. Natl. Acad. Sci. U.S.A. 2007; 104:18613–18618. 18003932 10.1073/pnas.0703637104 PMC2141825

[15] Gifford W.D., Pfaff S.L., Macfarlan T.S. Transposable elements as genetic regulatory substrates in early development. Trends Cell Biol. 2013; 23:218–226. 23411159 10.1016/j.tcb.2013.01.001 PMC4034679

[16] Wang J., Xie G., Singh M., Ghanbarian A.T., Raskó T., Szvetnik A., Cai H., Besser D., Prigione A., Fuchs N. V. et al. Primate-specific endogenous retrovirus-driven transcription defines naive-like stem cells. Nature. 2014; 516:405–409. 25317556 10.1038/nature13804

[17] Ecco G., Cassano M., Kauzlaric A., Duc J., Coluccio A., Offner S., Imbeault M., Rowe H.M., Turelli P., Trono D. Transposable elements and their KRAB-ZFP controllers regulate gene expression in adult tissues. Dev. Cell. 2016; 36:611–623. 27003935 10.1016/j.devcel.2016.02.024 PMC4896391

[18] Imbeault M., Helleboid P.-Y., Trono D. KRAB zinc-finger proteins contribute to the evolution of gene regulatory networks. Nature. 2017; 543:550–554. 28273063 10.1038/nature21683

[19] Wolf G., Yang P., Füchtbauer A.C., Füchtbauer E.-M., Silva A.M., Park C., Wu W., Nielsen A.L., Pedersen F.S., Macfarlan T.S. The KRAB zinc finger protein ZFP809 is required to initiate epigenetic silencing of endogenous retroviruses. Genes Dev. 2015; 29:538–554. 25737282 10.1101/gad.252767.114 PMC4358406

[20] Jacobs F.M.J., Greenberg D., Nguyen N., Haeussler M., Ewing A.D., Katzman S., Paten B., Salama S.R., Haussler D. An evolutionary arms race between KRAB zinc-finger genes ZNF91/93 and SVA/L1 retrotransposons. Nature. 2014; 516:242–245. 25274305 10.1038/nature13760 PMC4268317

[21] Slotkin R.K., Martienssen R. Transposable elements and the epigenetic regulation of the genome. Nat. Rev. Genet. 2007; 8:272–285. 17363976 10.1038/nrg2072

[22] Belancio V.P., Roy-Engel A.M., Deininger P.L. All y’all need to know ’bout retroelements in cancer. Semin. Cancer Biol. 2010; 20:200–210. 20600922 10.1016/j.semcancer.2010.06.001 PMC2943028

[23] Burns K.H. Transposable elements in cancer. Nat. Rev. Cancer. 2017; 17:415–424. 28642606 10.1038/nrc.2017.35

[24] Babaian A., Mager D.L. Endogenous retroviral promoter exaptation in human cancer. Mob. DNA. 2016; 7:24. 27980689 10.1186/s13100-016-0080-x PMC5134097

[25] Muotri A.R., Marchetto M.C.N., Coufal N.G., Oefner R., Yeo G., Nakashima K., Gage F.H. L1 retrotransposition in neurons is modulated by MeCP2. Nature. 2010; 468:443–446. 21085180 10.1038/nature09544 PMC3059197

[26] Li W., Jin Y., Prazak L., Hammell M., Dubnau J. Transposable elements in TDP-43-Mediated neurodegenerative disorders. PLoS One. 2012; 7:e44099. 22957047 10.1371/journal.pone.0044099 PMC3434193

[27] Larsen P.A., Hunnicutt K.E., Larsen R.J., Yoder A.D., Saunders A.M. Warning SINEs: Alu elements, evolution of the human brain, and the spectrum of neurological disease. Chromosom. Res. 2018; 26:93–111. 10.1007/s10577-018-9573-4 PMC5857278 29460123

[28] Larsen P.A., Lutz M.W., Hunnicutt K.E., Mihovilovic M., Saunders A.M., Yoder A.D., Roses A.D. The Alu neurodegeneration hypothesis: A primate-specific mechanism for neuronal transcription noise, mitochondrial dysfunction, and manifestation of neurodegenerative disease. Alzheimer's Dement. 2017; 13:828–838. 28242298 10.1016/j.jalz.2017.01.017 PMC6647845

[29] Ambati J., Fowler B.J. Mechanisms of age-related macular degeneration. Neuron. 2012; 75:26–39. 22794258 10.1016/j.neuron.2012.06.018 PMC3404137

[30] Newkirk S.J., Lee S., Grandi F.C., Gaysinskaya V., Rosser J.M., Vanden Berg N., Hogarth C.A., Marchetto M.C.N., Muotri A.R., Griswold M.D. et al. Intact piRNA pathway prevents L1 mobilization in male meiosis. Proc. Natl. Acad. Sci. U.S.A. 2017; 114:E5635–E5644. 28630288 10.1073/pnas.1701069114 PMC5514719

[31] Brennecke J., Malone C.D., Aravin A.A., Sachidanandam R., Stark A., Hannon G.J. An epigenetic role for maternally inherited piRNAs in transposon silencing. Science. 2008; 322:1387–1392. 19039138 10.1126/science.1165171 PMC2805124

[32] Houwing S., Kamminga L.M., Berezikov E., Cronembold D., Girard A., van den Elst H., Filippov D. V., Blaser H., Raz E., Moens C.B. et al. A role for Piwi and piRNAs in germ cell maintenance and transposon silencing in zebrafish. Cell. 2007; 129:69–82. 17418787 10.1016/j.cell.2007.03.026

[33] Malki S., van der Heijden G.W., O’Donnell K.A., Martin S.L., Bortvin A. A role for retrotransposon LINE-1 in fetal oocyte attrition in mice. Dev. Cell. 2014; 29:521–533. 24882376 10.1016/j.devcel.2014.04.027 PMC4056315

[34] Huang C.R.L., Burns K.H., Boeke J.D. Active transposition in genomes. Annu. Rev. Genet. 2012; 46:651–675. 23145912 10.1146/annurev-genet-110711-155616 PMC3612533

[35] Burns K.H., Boeke J.D. Human transposon tectonics. Cell. 2012; 149:740–752. 22579280 10.1016/j.cell.2012.04.019 PMC3370394

[36] Wicker T., Sabot F., Hua-Van A., Bennetzen J.L., Capy P., Chalhoub B., Flavell A., Leroy P., Morgante M., Panaud O. et al. A unified classification system for eukaryotic transposable elements. Nat. Rev. Genet. 2007; 8:973–982. 17984973 10.1038/nrg2165

[37] Stewart C., Kural D., Strömberg M.P., Walker J.A., Konkel M.K., Stütz A.M., Urban A.E., Grubert F., Lam H.Y.K., Lee W.P. et al. A comprehensive map of mobile element insertion polymorphisms in humans. PLoS Genet. 2011; 7:e1002236. 21876680 10.1371/journal.pgen.1002236 PMC3158055

[38] Abecasis G.R., Auton A., Brooks L.D., DePristo M.A., Durbin R.M., Handsaker R.E., Kang H.M., Marth G.T., McVean G.A. An integrated map of genetic variation from 1,092 human genomes. Nature. 2012; 491:56–65. 23128226 10.1038/nature11632 PMC3498066

[39] Giordano J., Ge Y., Gelfand Y., Abrusán G., Benson G., Warburton P.E. Evolutionary history of mammalian transposons determined by genome-wide defragmentation. PLoS Comput. Biol. 2007; 3:e137. 17630829 10.1371/journal.pcbi.0030137 PMC1914374

[40] Criscione S.W., Zhang Y., Thompson W., Sedivy J.M., Neretti N. Transcriptional landscape of repetitive elements in normal and cancer human cells. BMC Genomics. 2014; 15:583. 25012247 10.1186/1471-2164-15-583 PMC4122776

[41] Jin Y., Tam O.H., Paniagua E., Hammell M. TEtranscripts: a package for including transposable elements in differential expression analysis of RNA-seq datasets. Bioinformatics. 2015; 31:3593–3599. 26206304 10.1093/bioinformatics/btv422 PMC4757950

[42] Lerat E., Fablet M., Modolo L., Lopez-Maestre H., Vieira C. TEtools facilitates big data expression analysis of transposable elements and reveals an antagonism between their activity and that of piRNA genes. Nucleic Acids Res. 2016; 45:gkw953. 10.1093/nar/gkw953 PMC5389681 28204592

[43] Philippe C., Vargas-Landin D.B., Doucet A.J., van Essen D., Vera-Otarola J., Kuciak M., Corbin A., Nigumann P., Cristofari G. Activation of individual L1 retrotransposon instances is restricted to cell-type dependent permissive loci. Elife. 2016; 5:e13926. 27016617 10.7554/eLife.13926 PMC4866827

[44] Deininger P., Morales M.E., White T.B., Baddoo M., Hedges D.J., Servant G., Srivastav S., Smither M.E., Concha M., DeHaro D.L. et al. A comprehensive approach to expression of L1 loci. Nucleic Acids Res. 2017; 45:e31. 27899577 10.1093/nar/gkw1067 PMC5389711

[45] Scott E.C., Gardner E.J., Masood A., Chuang N.T., Vertino P.M., Devine S.E. A hot L1 retrotransposon evades somatic repression and initiates human colorectal cancer. Genome Res. 2016; 26:745–755. 27197217 10.1101/gr.201814.115 PMC4889970

[46] Krug L., Chatterjee N., Borges-Monroy R., Hearn S., Liao W.-W., Morrill K., Prazak L., Rozhkov N., Theodorou D., Hammell M. et al. Retrotransposon activation contributes to neurodegeneration in a Drosophila TDP-43 model of ALS. PLOS Genet. 2017; 13:e1006635. 28301478 10.1371/journal.pgen.1006635 PMC5354250

[47] Dobin A., Davis C.A., Schlesinger F., Drenkow J., Zaleski C., Jha S., Batut P., Chaisson M., Gingeras T.R. STAR: ultrafast universal RNA-seq aligner. Bioinformatics. 2013; 29:15–21. 23104886 10.1093/bioinformatics/bts635 PMC3530905

[48] Quinlan A.R., Hall I.M. BEDTools: a flexible suite of utilities for comparing genomic features. Bioinformatics. 2010; 26:841–842. 20110278 10.1093/bioinformatics/btq033 PMC2832824

[49] Li H., Handsaker B., Wysoker A., Fennell T., Ruan J., Homer N., Marth G., Abecasis G., Durbin R. 1000 Genome Project Data Processing Subgroup, 1000 Genome Project Data Processing The Sequence Alignment/Map format and SAMtools. Bioinformatics. 2009; 25:2078–2079. 19505943 10.1093/bioinformatics/btp352 PMC2723002

[50] Pertea M., Pertea G.M., Antonescu C.M., Chang T.-C., Mendell J.T., Salzberg S.L. StringTie enables improved reconstruction of a transcriptome from RNA-seq reads. Nat. Biotechnol. 2015; 33:290–295. 25690850 10.1038/nbt.3122 PMC4643835

[51] Love M.I., Huber W., Anders S. Moderated estimation of fold change and dispersion for RNA-seq data with DESeq2. Genome Biol. 2014; 15:550. 25516281 10.1186/s13059-014-0550-8 PMC4302049

[52] R Development Core Team, R R: A language and environment for statistical computing. R Found. Stat. Comput. 2011; 1:409.

[53] Li B., Ruotti V., Stewart R.M., Thomson J.A., Dewey C.N. RNA-Seq gene expression estimation with read mapping uncertainty. Bioinformatics. 2010; 26:493–500. 20022975 10.1093/bioinformatics/btp692 PMC2820677

[54] Huber W., Carey V.J., Gentleman R., Anders S., Carlson M., Carvalho B.S., Bravo H.C., Davis S., Gatto L., Girke T. et al. Orchestrating high-throughput genomic analysis with Bioconductor. Nat. Methods. 2015; 12:115–121. 25633503 10.1038/nmeth.3252 PMC4509590

[55] Taylor M.S., LaCava J., Mita P., Molloy K.R., Huang C.R.L., Li D., Adney E.M., Jiang H., Burns K.H., Chait B.T. et al. Affinity proteomics reveals human host factors implicated in discrete stages of LINE-1 retrotransposition. Cell. 2013; 155:1034–1048. 24267889 10.1016/j.cell.2013.10.021 PMC3904357

[56] Brawand D., Soumillon M., Necsulea A., Julien P., Csárdi G., Harrigan P., Weier M., Liechti A., Aximu-Petri A., Kircher M. et al. The evolution of gene expression levels in mammalian organs. Nature. 2011; 478:343–348. 22012392 10.1038/nature10532

[57] Smit AFA, Hubley R, Green P RepeatMasker Open-4.0. 2013–2015 http://www.repeatmasker.org.

[58] Langmead B., Trapnell C., Pop M., Salzberg S.L. Ultrafast and memory-efficient alignment of short DNA sequences to the human genome. Genome Biol. 2009; 10:R25. 19261174 10.1186/gb-2009-10-3-r25 PMC2690996

[59] Langmead B., Salzberg S.L. Fast gapped-read alignment with Bowtie 2. Nat. Methods. 2012; 9:357–359. 22388286 10.1038/nmeth.1923 PMC3322381

[60] Estes P.S., Daniel S.G., McCallum A.P., Boehringer A.V., Sukhina A.S., Zwick R.A., Zarnescu D.C. Motor neurons and glia exhibit specific individualized responses to TDP-43 expression in a Drosophila model of amyotrophic lateral sclerosis. Dis. Model. Mech. 2013; 6:721–733. 23471911 10.1242/dmm.010710 PMC3634655

[61] Marlor R.L., Parkhurst S.M., Corces V.G. The Drosophila melanogaster gypsy transposable element encodes putative gene products homologous to retroviral proteins. Mol. Cell. Biol. 1986; 6:1129–1134. 3023871 10.1128/mcb.6.4.1129-1134.1986 PMC367623

[62] Mejlumian L., Pélisson A., Bucheton A., Terzian C. Comparative and functional studies of Drosophila species invasion by the gypsy endogenous retrovirus. Genetics. 2002; 160:201–209. 11805056 10.1093/genetics/160.1.201 PMC1461946

[63] Kent W.J., Sugnet C.W., Furey T.S., Roskin K.M., Pringle T.H., Zahler A.M., Haussler D. The human genome browser at UCSC. Genome Res. 2002; 12:996–1006. 12045153 10.1101/gr.229102 PMC186604

[64] Kent W.J. BLAT–the BLAST-like alignment tool. Genome Res. 2002; 12:656–664. 11932250 10.1101/gr.229202 PMC187518

[65] Pruitt K.D., Brown G.R., Hiatt S.M., Thibaud-Nissen F., Astashyn A., Ermolaeva O., Farrell C.M., Hart J., Landrum M.J., McGarvey K.M. et al. RefSeq: an update on mammalian reference sequences. Nucleic Acids Res. 2014; 42:D756–D763. 24259432 10.1093/nar/gkt1114 PMC3965018

[66] Robinson J.T., Thorvaldsdóttir H., Winckler W., Guttman M., Lander E.S., Getz G., Mesirov J.P. Integrative genomics viewer. Nat. Biotechnol. 2011; 29:24–26. 21221095 10.1038/nbt.1754 PMC3346182

[67] Beck C.R., Garcia-Perez J.L., Badge R.M., Moran J. V LINE-1 elements in structural variation and disease. Annu. Rev. Genomics Hum. Genet. 2011; 12:187–215. 21801021 10.1146/annurev-genom-082509-141802 PMC4124830

[68] Deininger P. Alu elements: know the SINEs. Genome Biol. 2011; 12:236. 22204421 10.1186/gb-2011-12-12-236 PMC3334610

[69] Hancks D.C., Kazazian H.H. Jr SVA retrotransposons: Evolution and genetic instability. Semin. Cancer Biol. 2010; 20:234–245. 20416380 10.1016/j.semcancer.2010.04.001 PMC2945828

[70] Saito T., Rehmsmeier M. The precision-recall plot is more informative than the roc plot when evaluating binary classifiers on imbalanced datasets. PLoS One. 2015; 10:e0118432. 25738806 10.1371/journal.pone.0118432 PMC4349800

[71] Schwahn U., Lenzner S., Dong J., Feil S., Hinzmann B., van Duijnhoven G., Kirschner R., Hemberger M., Bergen A.A.B., Rosenberg T. et al. Positional cloning of the gene for X-linked retinitis pigmentosa 2. Nat. Genet. 1998; 19:327–332. 9697692 10.1038/1214

[72] Kimberland M.L., Divoky V., Prchal J., Schwahn U., Berger W., Kazazian H.H. Full-Length human L1 insertions retain the capacity for high frequency retrotransposition in cultured cells. Hum. Mol. Genet. 1999; 8:1557–1560. 10401005 10.1093/hmg/8.8.1557

[73] Smit A.F.A., Tóth G., Riggs A.D., Jurka J. Ancestral, Mammalian-wide subfamilies of LINE-1 repetitive sequences. J. Mol. Biol. 1995; 246:401–417. 7877164 10.1006/jmbi.1994.0095

[74] Boissinot S., Chevret P., Furano A. V. L1 (LINE-1) retrotransposon evolution and amplification in recent human history. Mol. Biol. Evol. 2000; 17:915–928. 10833198 10.1093/oxfordjournals.molbev.a026372

[75] Lee J., Cordaux R., Han K., Wang J., Hedges D.J., Liang P., Batzer M.A. Different evolutionary fates of recently integrated human and chimpanzee LINE-1 retrotransposons. Gene. 2007; 390:18–27. 17055192 10.1016/j.gene.2006.08.029 PMC1847406

[76] Upton K.R., Gerhardt D.J., Jesuadian J.S., Richardson S.R., Sánchez-Luque F.J., Bodea G.O., Ewing A.D., Salvador-Palomeque C., van der Knaap M.S., Brennan P.M. et al. Ubiquitous L1 mosaicism in hippocampal neurons. Cell. 2015; 161:228–239. 25860606 10.1016/j.cell.2015.03.026 PMC4398972

[77] Rodić N., Steranka J.P., Makohon-Moore A., Moyer A., Shen P., Sharma R., Kohutek Z.A., Huang C.R., Ahn D., Mita P. et al. Retrotransposon insertions in the clonal evolution of pancreatic ductal adenocarcinoma. Nat. Med. 2015; 21:1060–1064. 26259033 10.1038/nm.3919 PMC4775273

[78] Iskow R.C., McCabe M.T., Mills R.E., Torene S., Pittard W.S., Neuwald A.F., Van Meir E.G., Vertino P.M., Devine S.E. Natural mutagenesis of human genomes by endogenous retrotransposons. Cell. 2010; 141:1253–1261. 20603005 10.1016/j.cell.2010.05.020 PMC2943760

[79] Ewing A.D., Kazazian H.H. Jr High-throughput sequencing reveals extensive variation in human-specific L1 content in individual human genomes. Genome Res. 2010; 20:1262–1270. 20488934 10.1101/gr.106419.110 PMC2928504

[80] Gardner E.J., Lam V.K., Harris D.N., Chuang N.T., Scott E.C., Pittard W.S., Mills R.E. 1000 Genomes Project Consortium, 1000 Genomes Project 1000 Genomes Project Consortium, 1000 Genomes Project Devine S.E. The Mobile Element Locator Tool (MELT): population-scale mobile element discovery and biology. Genome Res. 2017; 27:1916–1929. 28855259 10.1101/gr.218032.116 PMC5668948

[81] Lee E., Iskow R., Yang L., Gokcumen O., Haseley P., Luquette L.J., Lohr J.G., Harris C.C., Ding L., Wilson R.K. et al. Landscape of somatic retrotransposition in human cancers. Science. 2012; 337:967–971. 22745252 10.1126/science.1222077 PMC3656569

[82] Keane T.M., Wong K., Adams D.J. RetroSeq: transposable element discovery from next-generation sequencing data. Bioinformatics. 2013; 29:389–390. 23233656 10.1093/bioinformatics/bts697 PMC3562067

[83] Sudmant P.H., Rausch T., Gardner E.J., Handsaker R.E., Abyzov A., Huddleston J., Zhang Y., Ye K., Jun G., Hsi-Yang Fritz M. et al. An integrated map of structural variation in 2,504 human genomes. Nature. 2015; 526:75–81. 26432246 10.1038/nature15394 PMC4617611

[84] Ewing A.D., Kazazian H.H. Jr Whole-genome resequencing allows detection of many rare LINE-1 insertion alleles in humans. Genome Res. 2011; 21:985–990. 20980553 10.1101/gr.114777.110 PMC3106331

[85] Gnanakkan V.P., Jaffe A.E., Dai L., Fu J., Wheelan S.J., Levitsky H.I., Boeke J.D., Burns K.H. TE-array–a high throughput tool to study transposon transcription. BMC Genomics. 2013; 14:869. 24325565 10.1186/1471-2164-14-869 PMC3878892

[86] Yue F., Cheng Y., Breschi A., Vierstra J., Wu W., Ryba T., Sandstrom R., Ma Z., Davis C., Pope B.D. et al. A comparative encyclopedia of DNA elements in the mouse genome. Nature. 2014; 515:355–364. 25409824 10.1038/nature13992 PMC4266106

[87] Scotter E.L., Chen H.-J., Shaw C.E. TDP-43 proteinopathy and ALS: Insights into disease mechanisms and therapeutic targets. Neurotherapeutics. 2015; 12:352–363. 25652699 10.1007/s13311-015-0338-x PMC4404432

[88] Maekawa S., Leigh P.N., King A., Jones E., Steele J.C., Bodi I., Shaw C.E., Hortobagyi T., Al-Sarraj S. TDP-43 is consistently co-localized with ubiquitinated inclusions in sporadic and Guam amyotrophic lateral sclerosis but not in familial amyotrophic lateral sclerosis with and without SOD1 mutations. Neuropathology. 2009; 29:672–683. 19496940 10.1111/j.1440-1789.2009.01029.x

[89] Chen-Plotkin A.S., Lee V.M.-Y., Trojanowski J.Q. TAR DNA-binding protein 43 in neurodegenerative disease. Nat. Rev. Neurol. 2010; 6:211–220. 20234357 10.1038/nrneurol.2010.18 PMC2892118

[90] Mackenzie I.R.A., Bigio E.H., Ince P.G., Geser F., Neumann M., Cairns N.J., Kwong L.K., Forman M.S., Ravits J., Stewart H. et al. Pathological TDP-43 distinguishes sporadic amyotrophic lateral sclerosis from amyotrophic lateral sclerosis withSOD1 mutations. Ann. Neurol. 2007; 61:427–434. 17469116 10.1002/ana.21147

[91] Diaper D.C., Adachi Y., Lazarou L., Greenstein M., Simoes F.A., Di Domenico A., Solomon D.A., Lowe S., Alsubaie R., Cheng D. et al. Drosophila TDP-43 dysfunction in glia and muscle cells cause cytological and behavioural phenotypes that characterize ALS and FTLD. Hum. Mol. Genet. 2013; 22:3883–3893. 23727833 10.1093/hmg/ddt243 PMC3766182

[92] Romano G., Appocher C., Scorzeto M., Klima R., Baralle F.E., Megighian A., Feiguin F. Glial TDP-43 regulates axon wrapping, GluRIIA clustering and fly motility by autonomous and non-autonomous mechanisms. Hum. Mol. Genet. 2015; 24:6134–6145. 26276811 10.1093/hmg/ddv330 PMC4599672

[93] Haidet-Phillips A.M., Hester M.E., Miranda C.J., Meyer K., Braun L., Frakes A., Song S., Likhite S., Murtha M.J., Foust K.D. et al. Astrocytes from familial and sporadic ALS patients are toxic to motor neurons. Nat. Biotechnol. 2011; 29:824–828. 21832997 10.1038/nbt.1957 PMC3170425

[94] Li W., Prazak L., Chatterjee N., Grüninger S., Krug L., Theodorou D., Dubnau J. Activation of transposable elements during aging and neuronal decline in Drosophila. Nat. Neurosci. 2013; 16:529–531. 23563579 10.1038/nn.3368 PMC3821974

[95] Tan H., Qurashi A., Poidevin M., Nelson D.L., Li H., Jin P. Retrotransposon activation contributes to fragile X premutation rCGG-mediated neurodegeneration. Hum. Mol. Genet. 2012; 21:57–65. 21940752 10.1093/hmg/ddr437 PMC3235010

[96] Song S.U., Gerasimova T., Kurkulos M., Boeke J.D., Corces V.G. An env-like protein encoded by a Drosophila retroelement: evidence that gypsy is an infectious retrovirus. Genes Dev. 1994; 8:2046–2057. 7958877 10.1101/gad.8.17.2046

[97] Li B., Dewey C.N. RSEM: accurate transcript quantification from RNA-Seq data with or without a reference genome. BMC Bioinformatics. 2011; 12:323. 21816040 10.1186/1471-2105-12-323 PMC3163565

[98] Beck C.R., Collier P., Macfarlane C., Malig M., Kidd J.M., Eichler E.E., Badge R.M., Moran J. V LINE-1 retrotransposition activity in human genomes. Cell. 2010; 141:1159–1170. 20602998 10.1016/j.cell.2010.05.021 PMC3013285

[99] Mir A.A., Philippe C., Cristofari G. euL1db: the European database of L1HS retrotransposon insertions in humans. Nucleic Acids Res. 2015; 43:D43–D47. 25352549 10.1093/nar/gku1043 PMC4383891

[100] Wang J., Song L., Grover D., Azrak S., Batzer M.A., Liang P. dbRIP: A highly integrated database of retrotransposon insertion polymorphisms in humans. Hum. Mutat. 2006; 27:323–329. 16511833 10.1002/humu.20307 PMC1855216

[101] Payer L.M., Steranka J.P., Yang W.R., Kryatova M., Medabalimi S., Ardeljan D., Liu C., Boeke J.D., Avramopoulos D., Burns K.H. Structural variants caused by Alu insertions are associated with risks for many human diseases. Proc. Natl. Acad. Sci. U.S.A. 2017; 114:E3984–E3992. 28465436 10.1073/pnas.1704117114 PMC5441760

[102] Faulkner G.J., Forrest A.R.R., Chalk A.M., Schroder K., Hayashizaki Y., Carninci P., Hume D.A., Grimmond S.M. A rescue strategy for multimapping short sequence tags refines surveys of transcriptional activity by CAGE. Genomics. 2008; 91:281–288. 18178374 10.1016/j.ygeno.2007.11.003

[103] Jeong H.-H., Yalamanchili H.K., Guo C., Shulman J.M., Liu Z. An ultra-fast and scalable quantification pipeline for transposable elements from next generation sequencing data. Biocomputing 2018. 2018; World Scientific 168–179. 29218879

[104] Brouha B., Schustak J., Badge R.M., Lutz-Prigge S., Farley A.H., Moran J.V., Kazazian H.H. Hot L1s account for the bulk of retrotransposition in the human population. Proc. Natl. Acad. Sci. U.S.A. 2003; 100:5280–5285. 12682288 10.1073/pnas.0831042100 PMC154336

[105] Tubio J.M.C., Li Y., Ju Y.S., Martincorena I., Cooke S.L., Tojo M., Gundem G., Pipinikas C.P., Zamora J., Raine K. et al. Mobile DNA in cancer. Extensive transduction of nonrepetitive DNA mediated by L1 retrotransposition in cancer genomes. Science. 2014; 345:1251343. 25082706 10.1126/science.1251343 PMC4380235

[106] Pitkänen E., Cajuso T., Katainen R., Kaasinen E., Välimäki N., Palin K., Taipale J., Aaltonen L.A., Kilpivaara O. Frequent L1 retrotranspositions originating from TTC28 in colorectal cancer. Oncotarget. 2014; 5:853–859. 24553397 10.18632/oncotarget.1781 PMC3996660

[107] Kalitsis P., Saffery R. Inherent promoter bidirectionality facilitates maintenance of sequence integrity and transcription of parasitic DNA in mammalian genomes. BMC Genomics. 2009; 10:498. 19860919 10.1186/1471-2164-10-498 PMC2777200

[108] Le T.N., Miyazaki Y., Takuno S., Saze H. Epigenetic regulation of intragenic transposable elements impacts gene transcription in Arabidopsis thaliana. Nucleic Acids Res. 2015; 43:3911–3921. 25813042 10.1093/nar/gkv258 PMC4417168

[109] Stower H. Alternative splicing: Regulating Alu element “exonization”. Nat. Rev. Genet. 2013; 14:152–153. 10.1038/nrg3428 23381119

[110] Sorek R., Ast G., Graur D. Alu-containing exons are alternatively spliced. Genome Res. 2002; 12:1060–1067. 12097342 10.1101/gr.229302 PMC186627

[111] Athanasiadis A., Rich A., Maas S. Widespread A-to-I RNA editing of Alu-containing mRNAs in the human transcriptome. PLoS Biol. 2004; 2:e391. 15534692 10.1371/journal.pbio.0020391 PMC526178

