# Supplementary material for: SQuIRE reveals locus-specific regulation of interspersed repeat expression
Source: Nucleic Acids Res. 2019 Jan 9;47(5):e27. doi: 10.1093/nar/gky1301 (PMC6411935; doi:10.1093/nar/gky1301)
Supplement: Supplementary Data [file gky1301_supplemental_files.pdf]

SUPPLEMENTARY METHODS

Implementation of STAR aligner in Map

Map uses parameters tailored to the alignment of TEs. By default, STAR only reports reads that map concordantly and to 10 or fewer locations. This setting discards reads that align to TEs that share sequence with more than 10 other copies. **Map** retains more reads mapped to TEs by reporting reads that fully map to 100 or fewer locations (--alignEndsType EndToEnd --outFilterMultimapNmax 100 –winAnchorMultimapNmax 100). For paired-end reads, **Map** also reports paired reads that map discordantly (--chimSegmentMin <read_length>) and single reads with unmapped mates or mates that were discarded for having too many alignments (--outFilterScoreMinOverLread 0.4 –outFilterMatchNminOverLread 0.4). These settings allow detection of RNA from TEs that are intrinsically expressed (often collinear and unspliced with few unique reads) as well as extrinsically expressed as part of larger transcripts (with unique reads overlapping neighboring sequence). **Map** can incorporate the non-reference TE sequences and generate a FASTA file that STAR adds to the genome index with the option "—genomeFastaFiles <fasta> ". To provide splicing information to the tools in the *Analysis Stage*, **Map** also uses the UCSC RefSeq gene annotation and assesses reads overlapping splice junctions with the options "—sjdbGTFfile <gtf> --sjdbOverhang <read_length -1> --twopassMode Basic". **Map** produces a sorted BAM file that includes intron and splicing information for downstream transcriptome assembly analysis.

Implementation of StringTie in Count for Gene Expression Quantification

Count runs StringTie [53] using these default settings guided by RefSeq gtf obtained from UCSC with **Fetch. Count** uses the "-e" StringTie option to quantify expression only to annotated transcripts without assembly of novel transcripts. StringTie outputs gene quantification as fpkm

and tpm by transcripts in a “gtf file” and by genes “abund.txt” file. We convert the fpkm values to counts by multiplying the per-exon coverage by exon length normalized by read length. StringTie quantifies reads that overlap with annotated exons; StringTie does not include completely intronic reads when run with the “-e” option in its fpkm calculation. If a TE-derived read overlaps with an annotated exon but the rest of the gene is not expressed, Stringtie reports gene expression in the abund.txt file, but the Stringtie gtf output identifies the transcript as a novel isoform (the source column is “Stringtie” instead of the reference gtf). If the TE-derived read overlaps with an intron and the rest of the gene is not expressed, there is no report of gene expression by Stringtie.

DESeq2 Implementation in Call

Call incorporates the Bioconductor package DESeq2 [54,55] with its suggested parameters. Users input the sample names and experimental design (ie which samples are treatment or control), which **Call** uses to find **Count** data and create a count matrix for annotated RefSeq genes and TEs. If the RNA-seq data is stranded, transcription from opposite strands of the same TE are treated separately. **Call** outputs differential expression tables and generates MA-plots, data quality assessment plots, and volcano plots.

STAR implementation in Draw

To visualize the distribution of reads across the TE, **Draw** runs STAR [52] with the parameters “–runMode input AlignmentsFromBAM –outWigType bedGraph” to provide visualization of read alignments. It will output bedgraphs of all reads (“multi”) and only uniquely (“unique”) aligning reads. **Draw** also compresses the bedgraphs into bigwig format for IGV [56] and UCSC Genome Browser [90] viewing. If the RNA-seq data is stranded it will output unique and multi bedgraphs for each strand.

Further details of Count

Count uses a combination of SAMTools (Li et al. 2009), BEDTools (Quinlan and Hall 2010), awk and bash within a Python script to perform the algorithm described in the main text, in particular distinguishing uniquely aligning reads from multi-mapping reads. Because the quantitation in SQuIRE relies on uniquely aligning reads, SQuIRE needed to resolve three issues in identifying uniquely aligning reads and their mapped TE location. 1) Because RepeatMasker annotation includes overlapping TE coordinates, a read can map uniquely at one genomic location corresponding to two TE loci. **Count** identifies these reads as unique by collapsing reads and their mapped TEs before labeling. The two TEs each would receive a unique count for that TE. 2) Similarly, when SQuIRE incorporates non-reference polymorphic TE insertions, its location can be confused with overlapping reference TE annotation. To address this, **Map** uses a custom chromosome name for non-reference TEs (eg. “chr3_poly”) during alignments. To keep read assignments to non-reference TEs separate from assignments to annotated TEs, **Count** changes the non-reference chromosome name back to the conventional name (eg “chr3”) only after collapsing reads mapped to the same location. III) For paired-end RNA-seq data, a read pair may map concordantly in only one location, particularly if one mate maps outside of the TE. However, one or both of the TE mapping mates may not be uniquely aligning, and map discordantly to other locations. In this situation, **Count** does not label these reads as “uniquely aligning”, but assigns a full count to the TE and discards the discordant alignments.

<table>
<tr><th>tx_chr</th><th>tx_start</th><th>tx_stop</th><th>TE_ID</th><th>fpkm</th><th>tx_strand</th><th>Sample</th><th>alignedsize</th><th>TE_chr</th><th>TE_start</th><th>TE_stop</th><th>TE_name</th><th>milliDiv</th><th>TE_strand</th><th>uniq_counts</th><th>tot_counts</th><th>tot_reads</th><th>score</th></tr>
<tr><td>chrX</td><td>150227860</td><td>150227918</td><td>chrX|150227860|150227918|Plat_L3:CR1:LINE|224|-</td><td>4228.95</td><td>+</td><td>sample1</td><td>10355420</td><td>chrX</td><td>150227860</td><td>150227918</td><td>Plat_L3:CR1:LINE</td><td>224</td><td>-</td><td>74</td><td>2539.97</td><td>2541</td><td>99.96</td></tr>
<tr><td>chr4</td><td>35784285</td><td>35784363</td><td>chr4|35784285|35784363|UCON49:L2:LINE|206|-</td><td>3415.7</td><td>+</td><td>sample1</td><td>10355420</td><td>chr4</td><td>35784285</td><td>35784363</td><td>UCON49:L2:LINE</td><td>206</td><td>-</td><td>112</td><td>2758.94</td><td>2759</td><td>100</td></tr>
<tr><td>chr14</td><td>94460277</td><td>94460382</td><td>chr14|94460277|94460382|L1ME4a:L1:LINE|233|+</td><td>2698.38</td><td>-</td><td>sample1</td><td>10355420</td><td>chr14</td><td>94460277</td><td>94460382</td><td>L1ME4a:L1:LINE</td><td>233</td><td>+</td><td>36</td><td>2934</td><td>2934</td><td>100</td></tr>
<tr><td>chr13</td><td>100961881</td><td>100962054</td><td>chr13|100961881|100962054|L2b:L2:LINE|283|-</td><td>2118.35</td><td>+</td><td>sample1</td><td>10355420</td><td>chr13</td><td>100961881</td><td>100962054</td><td>L2b:L2:LINE</td><td>283</td><td>-</td><td>132</td><td>3795</td><td>3795</td><td>100</td></tr>
<tr><td>chr22</td><td>38983462</td><td>38983650</td><td>chr22|38983462|38983650|MIR:MIR:SINE|319|+</td><td>1984.78</td><td>-</td><td>sample1</td><td>10355420</td><td>chr22</td><td>38983462</td><td>38983650</td><td>MIR:MIR:SINE</td><td>319</td><td>+</td><td>44</td><td>3864</td><td>3864</td><td>100</td></tr>
<tr><td>chr3</td><td>176423408</td><td>176423622</td><td>chr3|176423458|176423572|L1M5:L1:LINE|225|+</td><td>1800.79</td><td>-</td><td>sample1</td><td>10355420</td><td>chr3</td><td>176423458</td><td>176423572</td><td>L1M5:L1:LINE</td><td>225</td><td>+</td><td>0</td><td>3990.67</td><td>3991</td><td>99.99</td></tr>
</table>

Supplementary Table S1. Example output from SQuIRE **Count**

tx_start = start position of left-most read aligning to TE
tx_stop = stop position of right-most read aligning to TE
TE_ID = unique ID concatenating RepeatMasker annotation (see below): coordinates, TE name, milliDiv, and annotated strand. Each TE_ID may have up to two entries if RNA-seq data is stranded, one for each transcribed strand
fpkm = fragments per kilobase transcribed length per million aligned fragments
tx strand = strand of TE transcription
alignedsize = number of fragments with valid unique or multi alignments
TE_start = annotated RepeatMasker start
TE_stop = annotated RepeatMasker stop
TE_strand = annotated RepeatMasker strand (orientation of TE insertion)
milliDiv = Base mismatches in parts per thousand (from RepeatMasker)
uniq_count = # uniquely aligning reads
tot_count = # uniquely aligning reads + sum of multimapping fractions aligned to TE
tot_reads = # multi-mapping reads aligned to TE
score = tot_count/tot_reads * 100, which approximates how likely the TE is expressed with at least the tot_count

Chromosome/ Vector	Insertion_ start	Insertion _stop	Strand	Subfamily:Family:Order	Insertion_Type: Polymorphism,Novel, Plasmid, Transgene	Left- Flank_Seq	Right- Flank_Seq	TE_Seq
DA_L1RP	70	6087	+	L1HS:L1:LINE	Plasmid	CGTTTAGTGAACCGTCAGATCTCTAGAAGCTGGGTACCAGCTGCTAGCAAGCTTGCTAGCGGCCGCGGGG	ATCCAGACATGATAAGATACATTGATGAGTTTGGACAAACCACAACTAGAATGCAGTGAAAAAAATGCTTTATTTGTGAAATTTGTGATGCTATTGCTTTATTTGTAACCATTATAAGCTGCAATAAACAAGTTAACAACAACAATTGCATTCATTTTATGTTTCAGGTTCAGGGGGAGGTGTGGGAGGTTTTTTAAAGCAAGTAAAACC	GGAGGAGCCAAGATGGCCGAATAGGAACAGCTCCGGTCTACAGCTCCCAGCGTGAGCGACGCAGAAGACGGTGATTTCTGCATTTCCATCTGAGGTACCGGGTTCATCTCACTAGGGAGTGCCAGACAGTGGGCGCAGGCCAGTGTGTGTGCGCACCGTGCGCGAGCCGAAGCAGGGCGAGGCATTGCCTCACCTGGGAAGCGCAAGGGGTCAGGGAGTTCCCTTTCCGAGTCAAAGAAAGGGGTGACGGACGCACCTGGAAAATCGGGTCACTCCCACCCGAATATTGCGCTTTTCAGACCGGCTTAAGAAACGGCGCACCACGAGACTATATCCCGCACCTGGCTCGGAGGGTCCTACGCCCACGGAATCTCGCTGATTGCTAGCACAGCAGTCTGAGATCAAACTGCAAGGCGGCAACGAGGCTGGGGGAGGGGCGCCCGCCATTGCCCAGGCTTGCTTAGGTAAACAAAGCAGCAGGGAAGCTCGAACTGGGTGGAGCCCACCACAGCTCAAGGAGGCCTGCCTGCCTCTGTAGGCTCCACCTCTGGGGGCAGGGCACAGACAAACAAAAAGACAGCAGTAACCTCTGCAGACTTAAGTGTCCCTGTCTGACAGCTTTGAAGAGAGCAGTGGTTCTCCCAGCACGCAGCTGGAGATCTGAGAACGGGCAGACTGCCTCCTCAAGTGGGTCCCTGACCCCTGACCCCCGAGCAGCCTAACTGGGAGGCACCCCCCAGCAGGGGCACACTGACACCTCACACGGCAGGGTATTCCAACAGACCTGCAGCTGAGGGTCCTGTCTGTTAGAAGGAAAACTAACAACCAGAAAGGACATCTACACCGAAAACCCATCTGTACATCACCATCATCAAAGACCAAAAGTAGATAAAACCACAAAGATGGGGAAAAAACAGAACAGAAAAACTGGAAACTCTAAAACGCAGAGCGCCTCTCCTCCTCCAAAGGAACGCAGTTCCTCACCAGCAACAGAACAAAGCTGGATGGAGAATGATTTTGATGAGCTGAGAGAAGAAGGCTTCAGACGATCAAATTACTCTGAGCTACGGGAGGACATTCAAACCAAAGGCAAAGAAGTTGAAAACTTTGAAAAAAATTTAGAAGAATGTATAACTAGAATAACCAATACAGAGAAGTGCTTAAAGGAGCTGATGGAGCTGAAAACCAAGGCTCGAGAACTACGTGAAGAATGCAGAAGCCTCAGGAGCCGATGCGATCAACTGGAAGAAAGGGTATCAGCAATGGAAGATGAAATGAATGAAATGAAGCGAGAAGGGAAGTTTAGAGAAAAAAGAATAAAAAGAAATGAGCAAAGCCTCCAAGAAATATGGGACTATGTGAAAAGACCAAATCTACGTCTGATTGGTGTACCTGAAAGTGATGTGGAGAATGGAACCAAGTTGGAAAACACTCTGCAGGATATTATCCAGGAGAACTTCCCCAATCTAGCAAGGCAGGCCAACGTTCAGATTCAGGAAATACAGAGAACGCCACAAAGATACTCCTCGAGAAGAGCAACTCCAAGACACATAATTGTCAGATTCACCAAAGTTGAAATGAAGGAAAAAATGTTAAGGGCAGCCAGAGAGAAAGGTCGGGTTACCCTCAAAGGAAAGCCCATCAGACTAACAGCGGATCTCTCGGCAGAAACCCTACAAGCCAGAAGAGAGTGGGGGCCAATATTCAACATTCTTAAAGAAAAGAATTTTCAACCCAGAATTTCATATCCAGCCAAACTAAGCTTCATAAGTGAAGGAGAAATAAAATACTTTATAGACAAGCAAATGTTGAGAGATTTTGTCACCACCAGGCCTGCCCTAAAAGAGCTCCTGAAGGAAGCGCTAAACATGGAAAGGAACAACCGGTACCAGCCGCTGCAAAATCATGCCAAAATGTAAAGACCATCAAGACTAGGAAGAAACTGCACCAACTAATGAGCAAAATCACCAGCTAACATCATAATGACAGGATCAACTTCACACATAACAATATTAACTTTAAATATAAATGGACTAAATTCTGCAATTAAAAGACACAGACTGGCAAGTTGGATAAAGAGTCAAGACCCATCAGTGTGCTGTATTCAGGAAACCCATCTCACGTGCAGAGACACACATAGGCTCAAAATAAAAGGATGGAGGAAGATCTACCAAGCCAATGGAAAACAAAAAAAGGCAGGGGTTGCAATCCTAGTCTCTGATAAAACAGACTTTAAACCAACAAAGATCAAAAGAGACAAAGAAGGCCATTACATAATGGTAAAGGGATCAATTCAACAAGAGGAGCTAACTATCCTAAATATTTATGCACCCAATACAGGAGCACCCAGATTCATAAAGCAAGTCCTCAGTGACCTACAAAGAGACTTAGACTCCCACACATTAATAATGGGAGACTTTAACACCCCACTGTCAACATTAGACAGATCAACGAGACAGAAAGTCA

Supplementary Table S2. Non-reference table used to add L1RP plasmid sequence for TE read alignment and count estimation

		% Observed/Expected (for i E-M iterations)					
TE order	TE name	i = 0		i = auto		E-M improvement (%)	
		Locus	Subfamily	Locus	Subfamily	Locus	Subfamily
SINE	AluYa5	54.11	90.32	64.41	90.32	10.3	0
	AluYa8	69.69	79.89	85.05	88.11	15.36	8.22
	AluYb8	50.53	83.81	57.88	93.53	7.35	9.72
	AluYb9	48.47	91.6	63.25	93.94	14.78	2.34
LINE	L1HS	52.83	70.6	63.93	72.21	11.1	1.61

Supplementary Table S3. % of simulated reads that were reported by SQuIRE (% Observed/Expected) for frequently active human TEs at the locus and subfamily level. % Observed/Expected is improved with the use of Expectation-Maximization (EM) algorithm until convergence ("auto" number of interations) compared to no EM iterations.

		(for i E-M iterations)			
TE order	TE name	i = 0		i = auto	
		TPR	PPV	TPR	PPV
SINE	AluYa5	68.75	91.67	85.22	82.42
	AluYa8	83.33	100	100	100
	AluYb8	65.7	85.19	89.66	81.3
	AluYb9	81.82	64.29	90	64.29
LINE	L1HS	100	62.86	100	78.57

Supplementary Table S4. True positive rate (TPR) and positive predictive value (PPV) of SQuIRE Count for recently active human TEs. The % TPR is the % of loci with ≥ 10 simulated reads which SQuIRE reports to have ≥ 10 read counts. This indicates what percentage of expressed loci are detected by SQuIRE. The %PPV is the % of loci with ≥ 10 SQuIRE reads counts that in fact have ≥ 10 simulated reads. This indicates what percentage of loci are reported to have false positive expression.

Aligner	Library	Library (Reads)	TP	FP	TPR	PPV
Bowtie1	Single-end	4668185	4280536	143	91.7	100
Bowtie1	Paired-end	9336370	5074922	53991	54.36	98.95
Bowtie2	Single-end	4668185	3487593	3187	74.71	99.91
Bowtie2	Paired-end	9336370	8260128	7620	88.47	99.91
STAR	Single-end	4668185	4469031	15302	95.73	99.66
STAR	Paired-end	9336370	9107935	20113	97.55	99.78

Supplementary Table S5. True positive rate (TPR) and positive predictive value (PPV) of identifying uniquely aligning reads with different aligners. TPR=% true positive uniquely aligning reads to total reads in the library. PPV=% true positive uniquely aligning reads to total reported uniquely aligning reads.

Supplemental Figure S1. Outcomes of alternative non-SQuIRE algorithms for locus-level quantification of TE expression. a) Schematic of RNA-seq example from Figure 2. b) Pipelines that use only uniquely mapping reads can underestimate TE expression and miss the expression of TEs without unique sequence. c) Assigning multi-mapping reads in proportion to the number of mapped loci results in underestimation of TE A and overestimation of TEs B and C. d) Expectation-Maximization (EM) re-assignment of multi-mapped read fractions normalizes total read count by the annotated length instead of transcribed length. Although TE A, TE B, and TE C have the same annotated lengths, TE A is transcribed beyond the annotation and TE C's transcript is shorter than annotated. Without this transcription information, EM continually overestimates the expression level of TE A and underestimates the expression of TE C, thereby assigning ever greater fractions to TE A and smaller fractions to TE C until convergence.

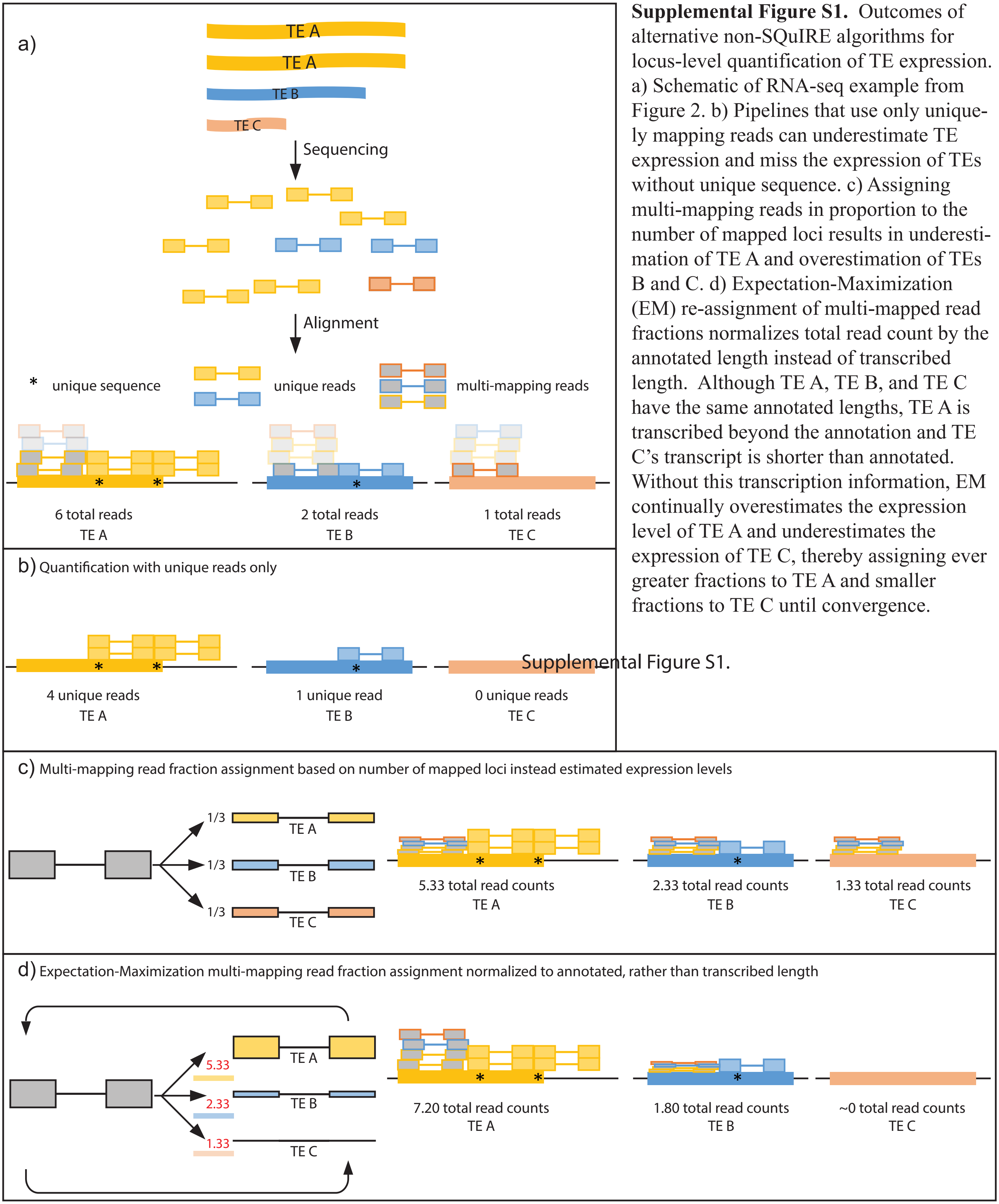

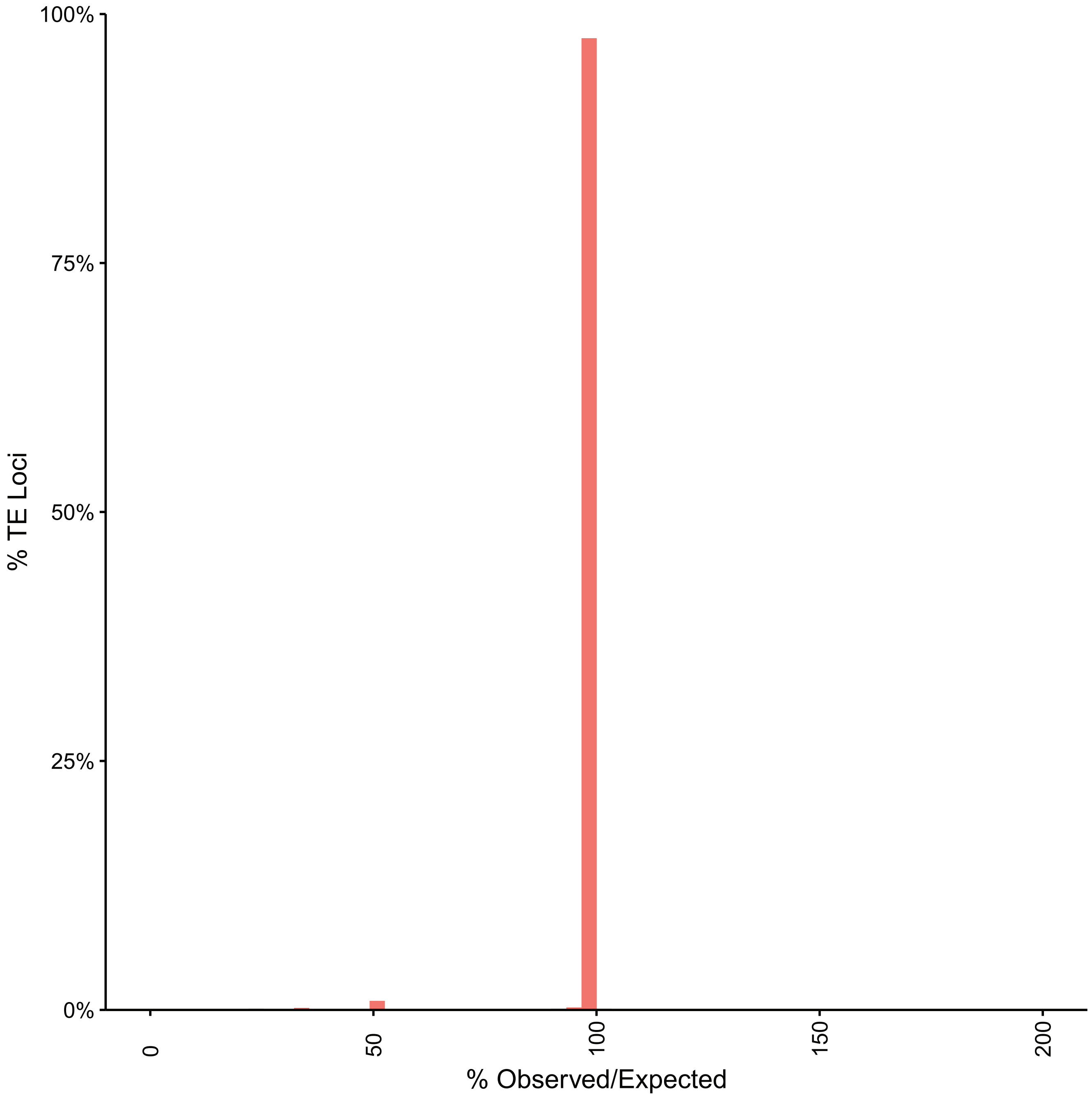

Supplemental Figure S2. Percent of simulated reads (Expected) included in SQuIRE read count output (Observed) for each TE. Histogram of % Observed/Expected as percentage of 100,000 simulated TEs.

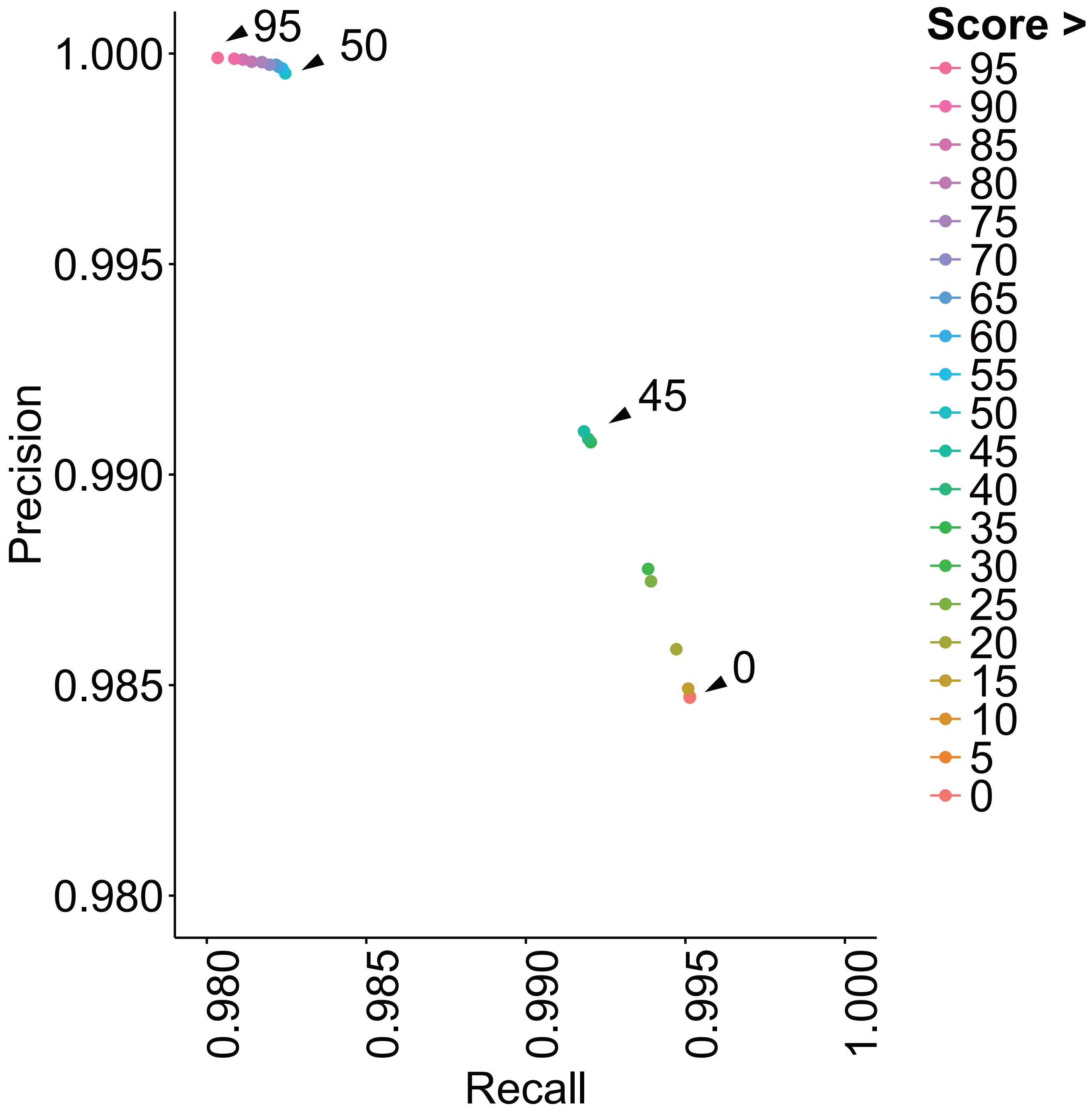

Supplemental Figure S3. Precision-Recall curve of SQuIRE Count with varying score thresholds. Precision= Σ "True positive"/ Σ "Positive". Recall=Σ "True positive"/ Σ "True". Positive=SQuIRE reported the TE has a count >10. True=TE was simulated to express > 10 reads. A score threshold of >50 maximizes precision and recall for the TEs in the hg38 genome.

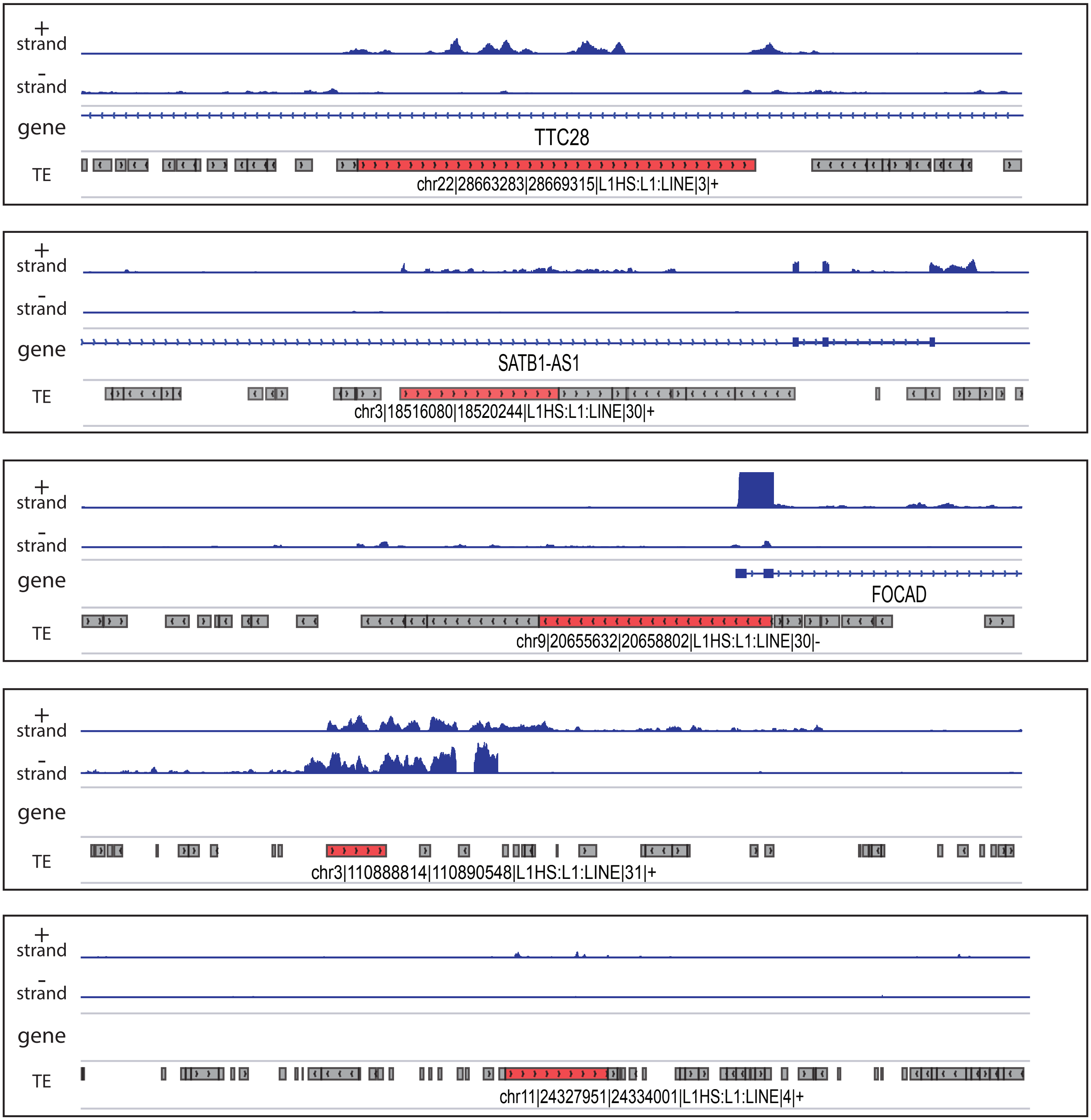

Supplemental Figure S4. L1HS loci detected in HEK293T cells that initiate at the 5' end of the L1HS locus (red boxes).

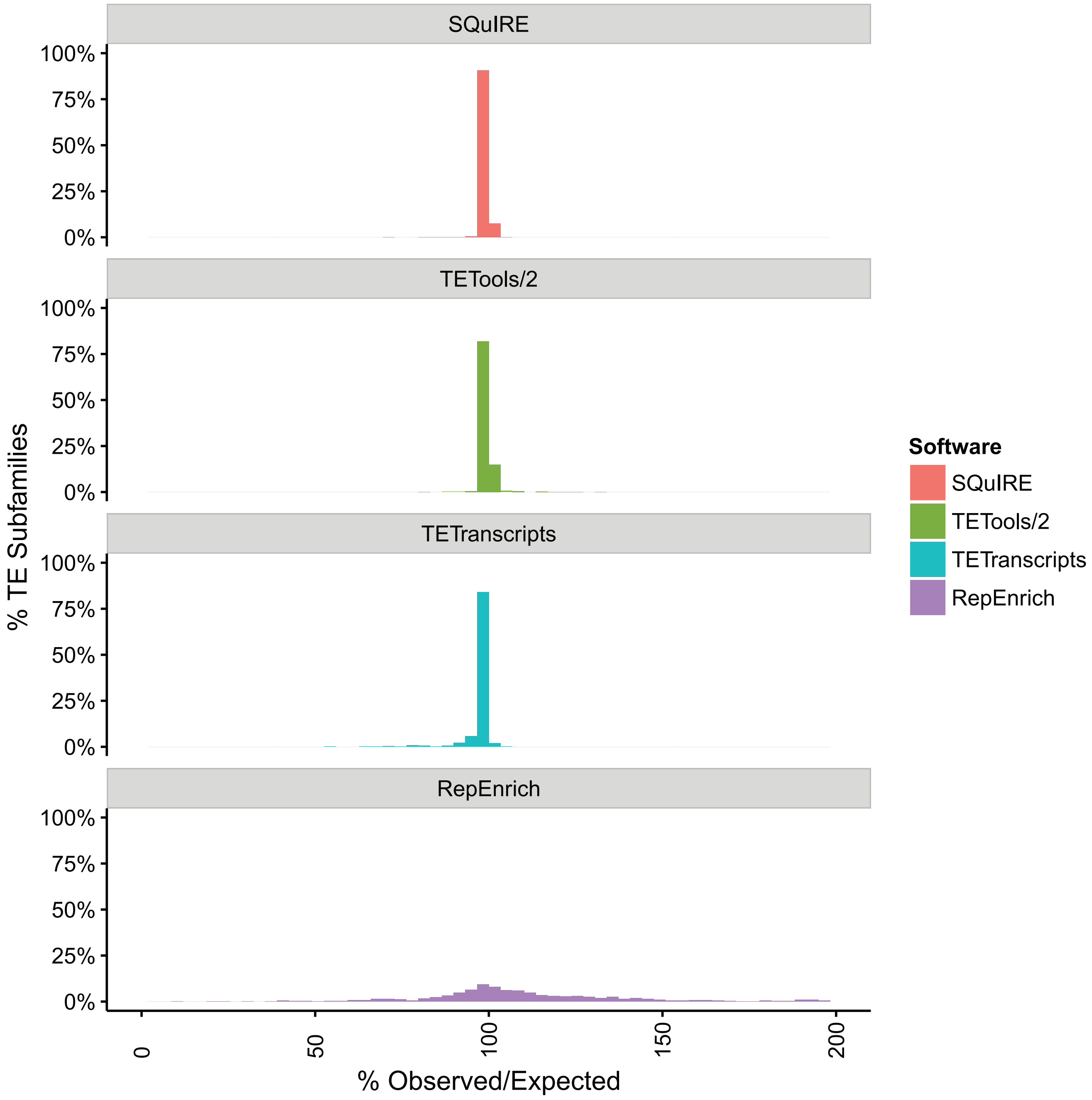

Supplemental Figure S5. Comparison of TE RNA-seq tools at the subfamily level for simulated data. Histogram of % TE subfamilies for each percentage of reported over simulated counts. SQuIRE has the tallest and narrowest peak near 100% Observed/Expected, indicating the it is correctly attributing simulated reads to the greatest number of subfamilies. Because TETools outputs in reads rather than fragments, its output is twice that of the other software.

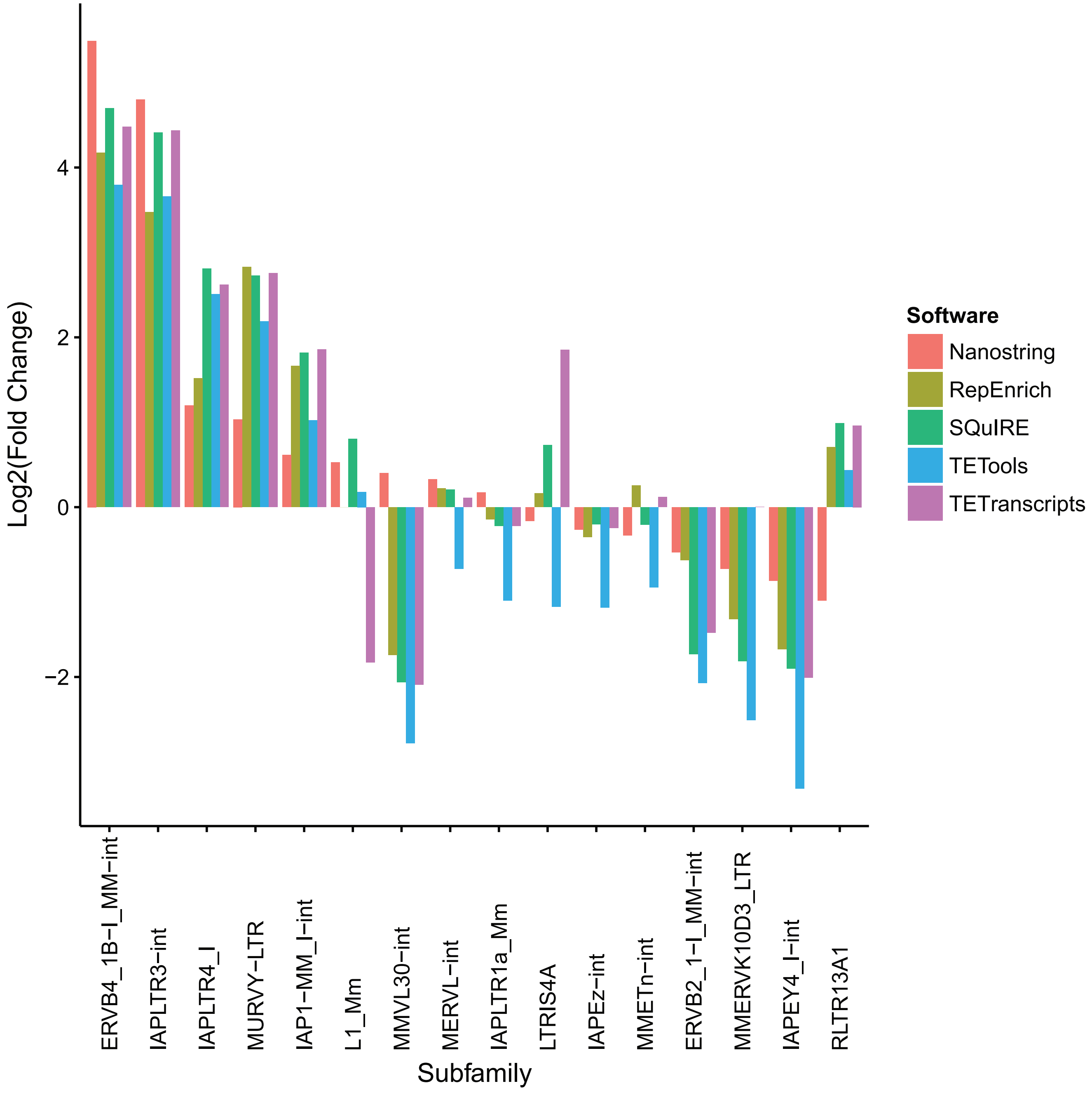

Supplemental Figure S6. Bar plot comparison of TE RNA-seq tools compared to Nanostring data at the subfamily level. Y-axis represents $\log_2$ fold changes of subfamily expression in testis compared to pooled somatic tissues (brain, heart, kidney, and liver).

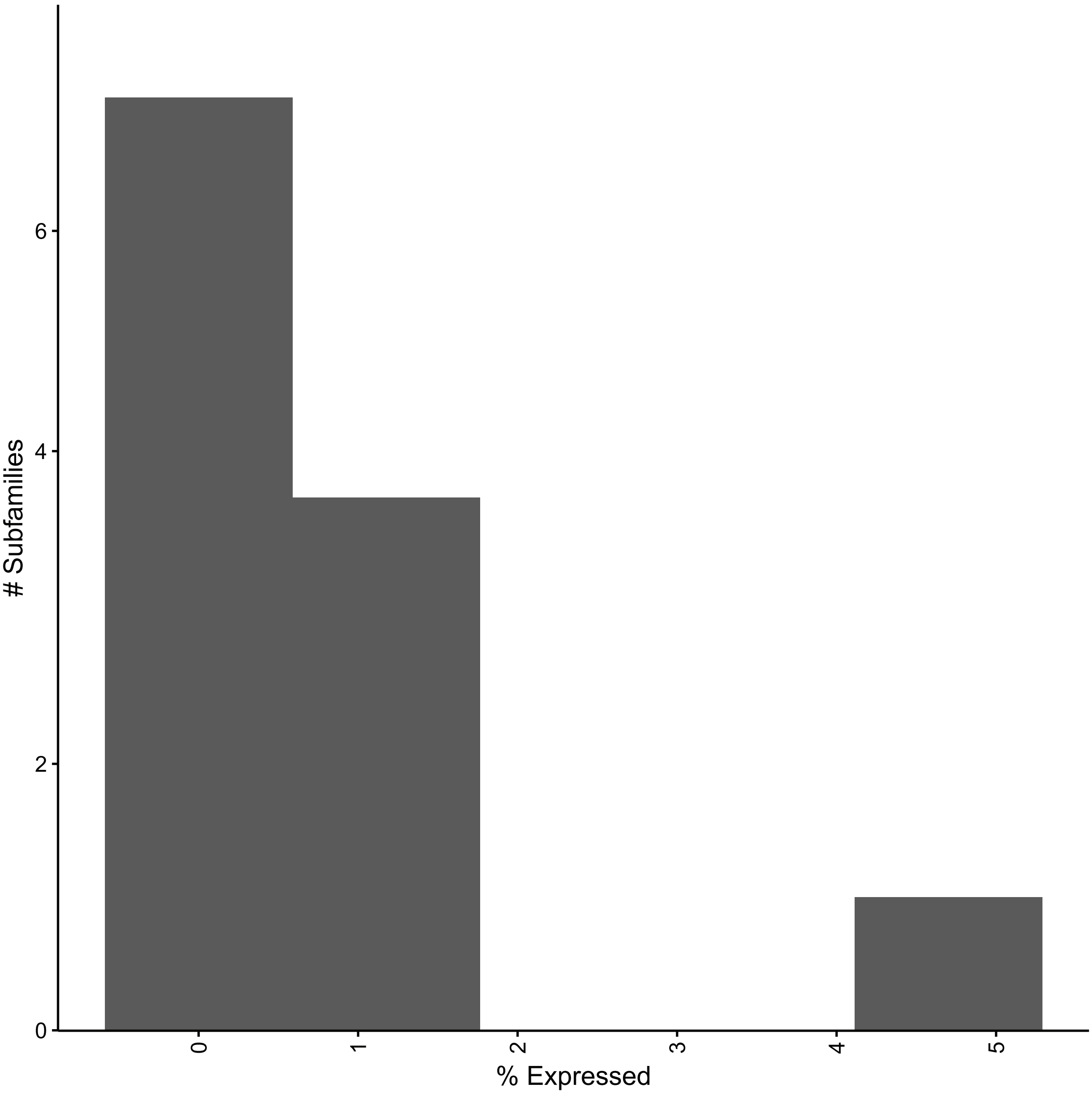

Supplemental Figure S7. Histogram showing a small percentage of loci for each mouse TE subfamily exhibit expression. X-axis represents percentage of loci expressed with greater than 10 counts. Y-axis represents number of subfamilies (among the 16 subfamilies analyzed in Supplemental Fig. S5).

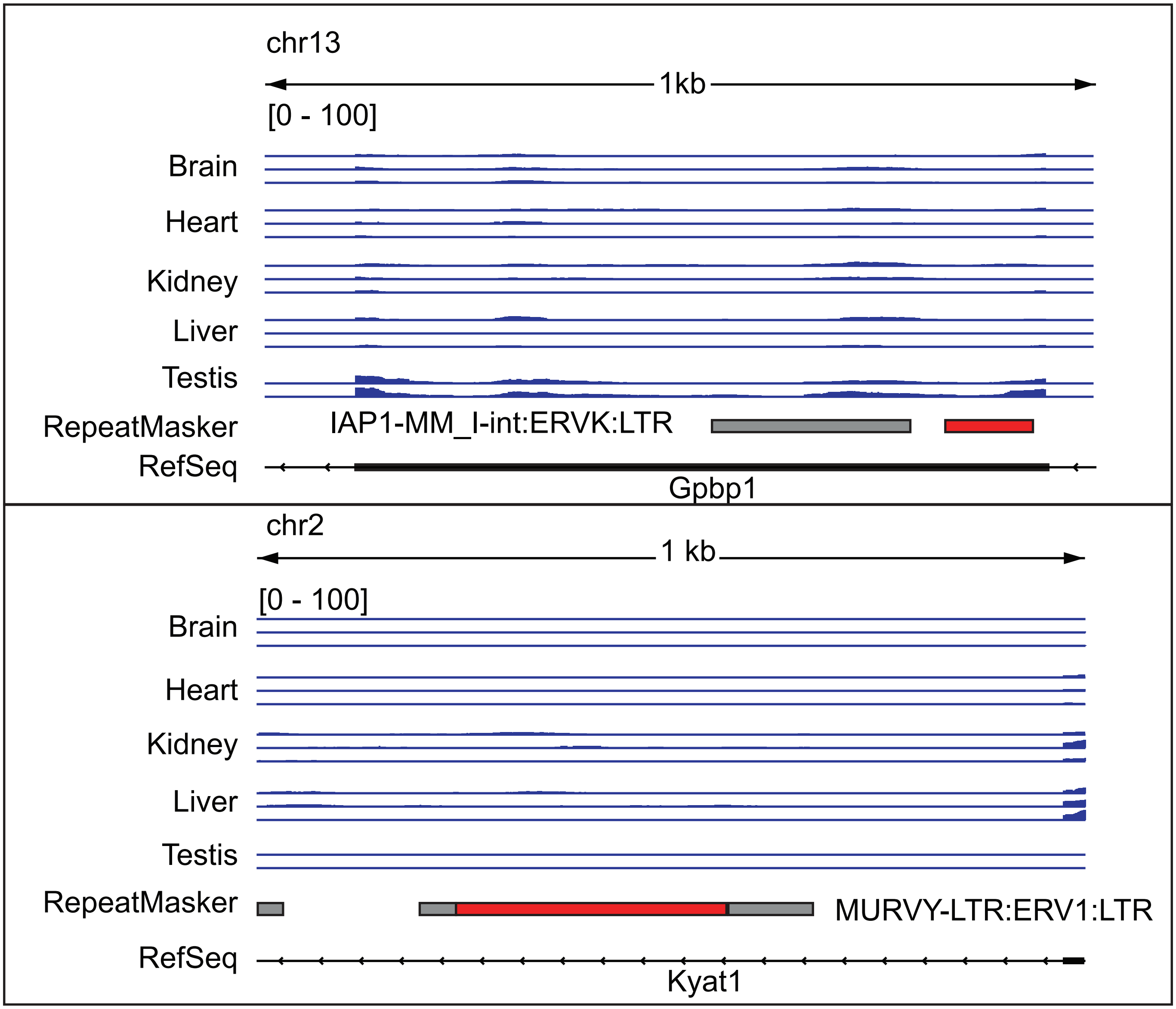

Supplemental Figure S8. Examples of intragenic TE loci differentially expressed in somatic tissues compared to testis. Replicates from brain, heart, kidney and liver are grouped in adjacent tracks. The scales of count expression are shown in brackets. The RepeatMasker track represents TEs annotated in the reference genome.The RefSeq track represents annotated genes. Transposable elements colored in red belong to the subfamily indicated; dark red indicates that the TE meets significant differential expression thresholds (log2FC > 2, padj < 0.05).
